# New determinant of influenza hemagglutinin cleavability identified through random mutagenesis for acid-stabilizing mutations

**DOI:** 10.1128/spectrum.04123-25

**Published:** 2026-06-15

**Authors:** S. Rimaux, M. F. Oliva, C. Mestdagh, R. Van Berwaer, J. Chen, L. Schurmans, G. Schoofs, B. Vanmechelen, J. Stroobants, M. Jacquemyn, M. Laporte, P. Maes, K. Vermeire, K. Das, A. Stevaert, L. Naesens

**Affiliations:** 1 Molecular, Structural and Translational Virology Research Group, Department of Microbiology, Immunology and Transplantation, Rega Institute, KU Leuven 573654 , Leuven, Belgium; 2 Laboratory of Clinical and Epidemiological Virology, Department of Microbiology, Immunology and Transplantation, Rega Institute, KU Leuven 573654 , Leuven, Belgium; 3 Molecular Genetics and Therapeutics in Virology and Oncology Research Group, Department of Microbiology, Immunology and Transplantation, Rega Institute, KU Leuven 573654 , Leuven, Belgium; 4 Virology, Antiviral Drug and Vaccine Research Group, Department of Microbiology, Immunology and Transplantation, Rega Institute, KU Leuven 573654 , Leuven, Belgium; 5 European Plotkin Institute for Vaccinology (EPIV), Université Libre de Bruxelles (ULB) 26659, Brussels, Belgium; Erasmus MC, Rotterdam, the Netherlands

**Keywords:** influenza, hemagglutinin, fusion, cleavage, stability, random mutagenesis

## Abstract

The structural instability of influenza hemagglutinin (HA) is related to its function in low pH-mediated membrane fusion, which requires prior cleavage of the premature HA0 by a host protease. Mapping the precise determinants of HA cleavability and stability has implications for risk assessment of zoonotic influenza A viruses, viral transmissibility, and vaccine production. Here, we conducted random mutagenesis on the early 2009 pandemic H1 HA, followed by selection of acid-stable viruses, and detailed profiling of the mutant HAs. We identified mutation D346N, which is located in the cleavage loop and renders H1 HA0 12-fold resistant to trypsin activation. Its cleavage by transmembrane serine protease 2 (TMPRSS2) was proved not to be affected. Likewise, we found that the poor cleavage of H16 HA0, which is unusual in carrying an N346 residue, is evident with exogenous trypsin and when non-activated particles encounter TMPRSS2 only during entry. Since H16 HA also exhibits a very low fusion pH, we propose that the gull H16N3 virus may carry a much more stable HA than other avian influenza A viruses. In addition, our random mutagenesis study on H1 HA yielded four acid-stabilizing mutations, which decrease the pH and efficiency of membrane fusion, and subtly impair the viral entry and replication in human airway-derived epithelial cells. These mutations are situated in the globular head, vestigial esterase, and membrane-proximal part of H1 HA, in regions involved in the refolding of HA at low pH. Collectively, our mutagenesis approach revealed determinants of HA cleavability and stability, with relevance for viral surveillance and vaccine production.

**IMPORTANCE:**

The presence of influenza A viruses (IAV) throughout the animal world, particularly avian species, represents a constant threat for zoonotic infections or a new influenza pandemic. To be transmissible among humans, a zoonotic IAV requires a hemagglutinin (HA) that is activated by host proteases and exhibits appropriate stability. Mapping the underlying determinants also matters for producing HA-based vaccines with high shelf stability. Through random mutagenesis and selection of acid-stable viruses, we discovered a mutation that renders HA resistant to exogenous trypsin activation. The natural occurrence of this residue in H16 HA, combined with the prominent acid stability of this HA subtype, suggests that the gull H16N3 virus may differ from other avian IAVs in carrying an environmentally stable HA. Besides, we identified four stabilizing mutations located in different parts of HA. Hence, our study delivers insight into factors that modulate HA cleavability and acid stability, with relevance for viral surveillance and vaccine production.

## INTRODUCTION

The propensity of influenza A virus (IAV) to cause zoonotic infections and sporadic pandemics in humans is related to the wide diversity of IAV subtypes that circulate in animals, particularly avian species (1). The last influenza pandemic occurred in 2009 and resulted from a swine-origin reassortant virus [A(H1N1)pdm09] that evolved into an annually recurring seasonal IAV (2). This pandemic spurred significant interest in understanding, at the molecular level, how a zoonotic IAV adapts to humans. Among the different viral factors involved (3), the hemagglutinin (HA) is a key player since it controls viral entry into the host cell. This process starts with viral binding to sialoglycans and uptake by endocytosis. The endosomal pH (~5) triggers drastic refolding of HA, which proceeds via several reversible states (4–6) and leads to extrusion of the fusion peptide. After fusion of the endosomal and viral membranes, the viral genome segments are released into the cytoplasm. The protein structures of pre- and post-fusion HA are dramatically different and resolved in quite many details (5, 7).

To acquire affinity for the upper part of the human respiratory tract, an avian IAV requires HA mutations that switch its tropism from α2,3- to α2,6-linked sialoglycans (8). Another adaptation mechanism relates to the ability of HA to undergo cleavage activation by human airway proteases (9–12) and, once cleaved, remain stable upon virus shedding in the environment (13, 14). In virus-infected cells, HA is initially synthesized as its HA0 precursor, which requires cleavage into the HA1/HA2 pre-fusion form that is fusion-competent yet metastable. Multibasic H5 and H7 HAs are activated by furin and other proprotein convertases (9). For monobasic HAs, diverse trypsin-like proteases (such as the membrane-anchored enzymes transmembrane serine protease 2 [TMPRSS2] and human airway trypsin-like protease [HAT]; [11]) have been implicated, with evidence for certain subtype dependency (10). For instance, the H13 and H16 HA0s proved exceptional in resisting exogenous trypsin (10), and for H16 HA0, this was attributed to an unusual α-helix that prevents access to the cleavage loop (15). Besides, HA0-cleaving proteases vary in their preference for amino acid residues preceding the scissile arginine (16, 17), as well as in their (sub)cellular location and functioning (18). TMPRSS2 was reported to accumulate in the Golgi apparatus and trans-Golgi network (TGN), while HAT was predominantly detected at the plasma membrane (18, 19).

The subtle association between HA stability and influenza virus transmissibility was first discovered in ferret models. In two studies focusing on H5 HA, the airborne-transmitted virus contained an HA mutation that increases the acid- and thermostability of HA (20, 21). Conversely, reduced transmission in ferrets was noted with a mutant A(H1N1)pdm09 virus that carried a less acid-stable HA than the wild-type (WT) virus (14). The association is supported by surveillance data in humans since, within 3 years after its emergence, the A(H1N1)pdm09 virus acquired HA mutations that increased the protein’s stability (14, 22, 23). Such mutations are also important for vaccine production, since they may increase viral growth (24) or the vaccine’s thermal stability and shelf-life (22, 24, 25). The list of known stabilizing HA mutations is quite extensive and steadily growing (13, 26, 27).

In the present study, we applied random mutagenesis to identify additional sites in HA that modulate its acid stability and cleavability. We chose the HA of an early A(H1N1)pdm09 isolate as our model protein, since its relatively high fusion pH (i.e., low acid-stability) makes it amenable to acquisition of stabilizing mutations. After selecting acid-stable viruses, we identified four mutations associated with a lower fusion pH, besides one mutation, D346N, which impaired the cleavage activation by trypsin. We presumed an analogy with the trypsin-resistant H16 HA0 (10, 15), which differs from most other subtypes in carrying an asparagine (N) at residue 346, and we therefore decided to include H16 HA in our study. Additionally, the acid-stabilizing mutations were thoroughly investigated for their impact on membrane fusion and viral entry. Finally, we interpreted our biological findings with respect to the protein structure of uncleaved or cleaved HA.

## RESULTS

### Random mutagenesis and selection of HA mutations conferring higher acid stability

**Fig 1:**
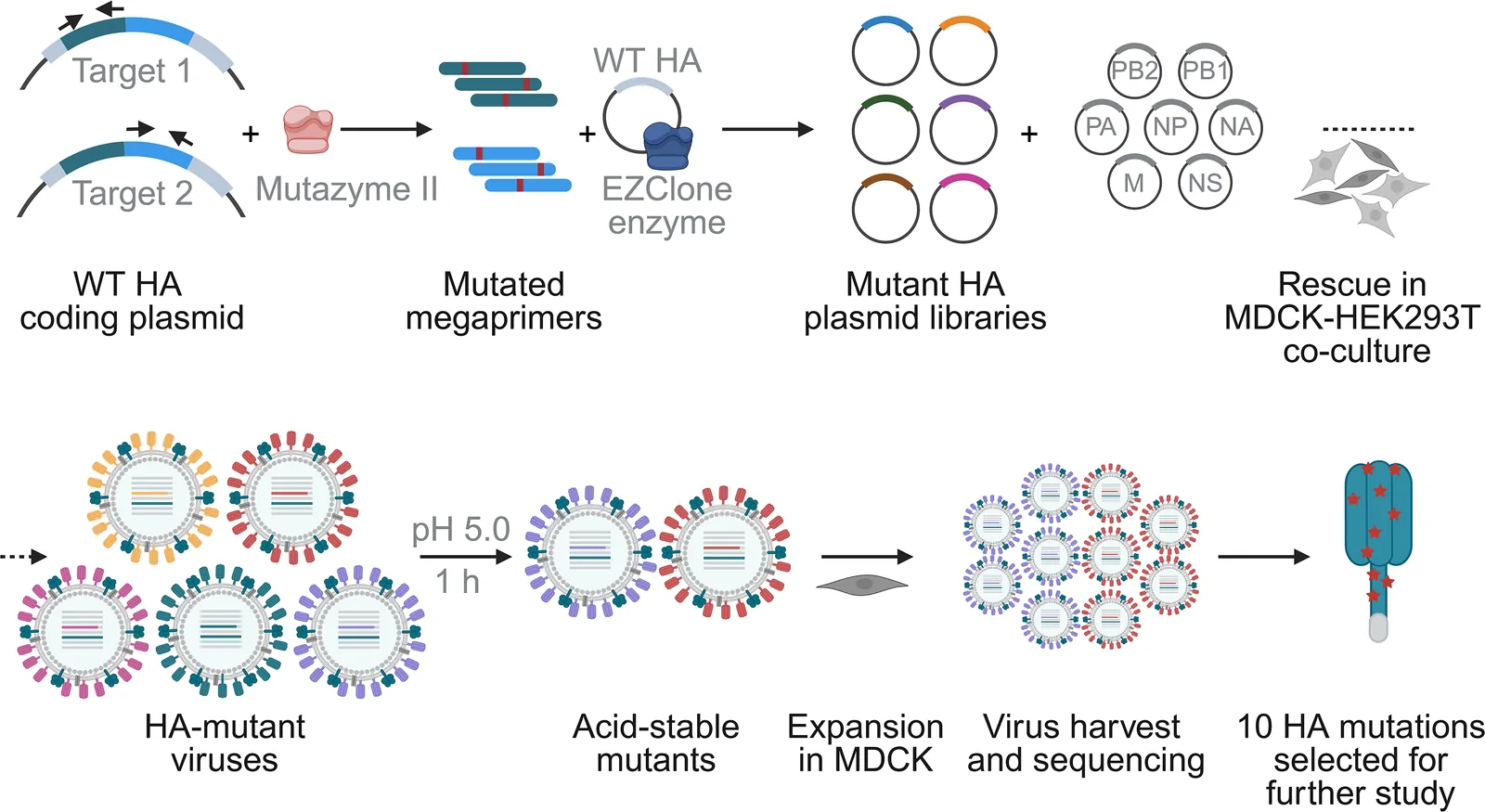
Procedure for random mutagenesis and selection of HA-mutant H1N1 viruses with higher acid stability. The reverse genetics plasmid encoding Virg09-HA was submitted to error-prone PCR and combined with the other seven plasmids to generate HA-mutant libraries of Virg09_PR8_ virus. After 1 h incubation at pH 5.0, the surviving mutants were expanded and sequenced. This yielded 10 HA mutations for follow-up.

To identify stabilizing mutations, we selected the HA of strain A/Virginia/ATCC3/2009 (Virg09), which was isolated soon after the start of the 2009 pandemic and carries an HA of intermediate stability (fusion pH ~ 5.5). After introducing this HA sequence in a reverse genetics plasmid, we conducted error-prone PCR (Fig. 1). The mutation rate was controlled by splitting the HA sequence into two parts, using two primer sets for amplification, and conducting the PCR with 500 ng input DNA and 30 PCR cycles. In this way, we generated six mutagenized HA plasmid libraries (i.e., two HA parts and three separate PCR reactions), which served to reverse-engineer six mutant libraries of Virg09_PR8_ virus (i.e., chimeric virus with internal genes of PR8, and HA and NA sequences of Virg09).

To select mutant viruses with a more acid-stable HA, the Virg09_PR8_ libraries were incubated for 1 h at pH 5.0 (37°C), then applied to Madin-Darby canine kidney (MDCK) cells to expand viruses that survived the low pH treatment (Fig. 1). Supernatants from wells showing viral cytopathic effect (CPE) were harvested for HA sequencing. Notably, we use the same H1 HA0 numbering as another study on HA-stabilizing mutations (22) (see Table S1 for the corresponding H3 numbering). This yielded ten mutations, which were introduced in an HA expression plasmid to produce mutant H1N1 pseudoviruses. Following release from HEK293T producer cells, the pseudoviruses were incubated with trypsin and first checked, by automated western blot, for proper HA expression and cleavage activation by trypsin. Quite remarkably, the D346N-mutant pseudovirions differed from all other nine mutants and the WT in carrying hardly any cleaved HA, indicating trypsin resistance (Fig. S1). Before characterizing the other mutations, we first investigated this unexpected finding in more depth.

### Mutation D346N impairs trypsin cleavage of H1 HA0, while N346D enhances cleavage of H16 HA0

**Fig 2:**
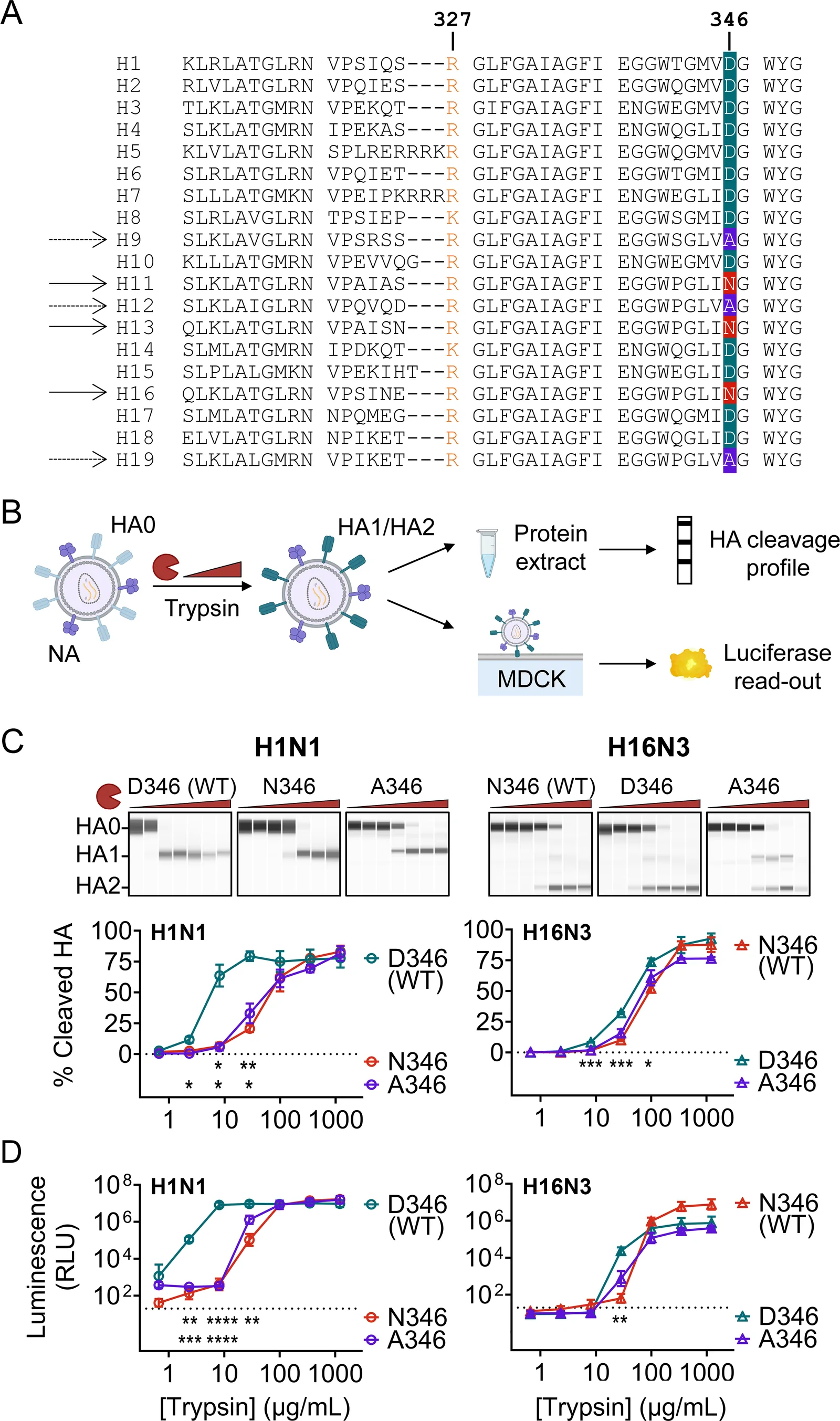
Cleavage loop variation and trypsin dependence of H1 and H16 HA0 proteins carrying D346, N346, or A346. (**A**) Alignment of the cleavage loop sequences of H1–H19 HA0. The scissile arginine is colored orange, and the residue at position 346 is boxed in teal (Asp; D), red (Asn; N), or purple (Ala; A). Dotted arrows indicate HA subtypes with A346, and full arrows indicate HA subtypes with N346. (**B–D**) Trypsin dependence of H1 HA and H16 HA bearing residues D346, N346, and A346. (**B**) HA0-bearing pseudoviruses were exposed to different concentrations of trypsin. (**C**) HA0 cleavage status was analyzed by Simple Western with antibodies recognizing HA1 (H1 HA) or HA1 and HA2 (H16 HA). Representative images and mean ± SEM (*N* = 3) of percent cleaved HA are shown. (**D**) The activated pseudoviruses were incubated with MDCK cells for 24 h, followed by luciferase readout. Data points are the mean relative luminescence unit (RLU) values ± SEM (*N* = 3–5). The dotted line represents the baseline without trypsin activation. Differences between WT and mutants were analyzed by an unpaired *t*-test with Holm-Šídák correction for multiple comparisons. ****P ≤ 0.0001, ***P ≤ 0.001, **P ≤ 0.01, and *P ≤ 0.05

Residue D346 is located in the cleavage loop of HA0, which requires cleavage at the scissile arginine (R327 based on the numbering used here) to liberate the fusion peptide, i.e., the N-terminal region of HA2. We first analyzed the conservation rate of residue D346 across the 19 IAV-HA subtypes. After generating a consensus sequence for each subtype, followed by alignment of all subtypes (Fig. 2A), we found that D346 is conserved in 13 of the 19 HAs. It is substituted by alanine (A) in the H9, H12, and H19 HAs, and by asparagine (N) in the H11, H13, and H16 HAs. The H13 and H16 proteins were reported to be trypsin resistant (10), and for H16 HA0, this was explained by the presence of a peculiar and short α-helix in the cleavage loop, as evident from crystallography (15). Hence, while investigating mutation D346N in H1 HA, we included the inverse mutation (N346D) in H16 HA, as well as the alanine variation at this position.

H1N1 and H16N3 pseudoviruses, bearing WT or mutant HA, were produced in HEK293T cells (experimental setup shown in Fig. 2B). After release, they were incubated with a range of trypsin concentrations (i.e., 3.5-fold serial dilutions centered around 100 µg/mL), to then analyze their HA0 cleavage status by simple western analysis (Fig. 2C; note that the anti-H1 antibody detected only the HA1 cleavage product, while the anti-H16 antibody recognized both HA1 and HA2). In parallel, we transduced the pseudoviruses into MDCK cells to measure their entry efficiency, which closely mirrored the HA0 cleavage results (Fig. 2D). As expected, H1^D346^ (WT) HA0 was efficiently cleaved, with a detectable HA1 band at 2 µg/mL of trypsin and ~60% cleaved protein at 8 µg/mL. In sharp contrast but consistent with previous reports (10, 15), the H16^N346^ (WT) protein required ~12-fold more trypsin (~100 µg/mL) to achieve ~50% cleavage (Fig. 2C). The reliance on high trypsin concentrations was also seen with the H1^N346^ and H1^A346^ mutant HAs, which showed a very similar behavior and required significantly more trypsin than the WT (H1^D346^) for HA0 cleavage and viral entry (Fig. 2C and D; curves in red and purple). Reciprocally, the H16^D346^ mutant showed a slight, yet significant, shift toward lower trypsin requirement when compared to the H16^N346^ (WT) and H16^A346^ forms. For instance, the H16^D346^ mutant showed minimal cleavage at 8 µg/mL of trypsin compared to 28 µg/mL for H16^N346^ (Fig. 2C).

Taken together, in H1 HA0, the trypsin-mediated activation is impaired by substituting residue D346 with an N or A. In H16 HA0, replacing N346 with a D residue enhances trypsin cleavage, partially compensating for the low activation efficiency of the H16 subtype. This points to an important role of residue 346 in modulating HA0 activation by trypsin.

### Cleavage by TMPRSS2 is not significantly affected by mutating residue 346 and, for H16 HA, is markedly more efficient in producer than target cells

**Fig 3:**
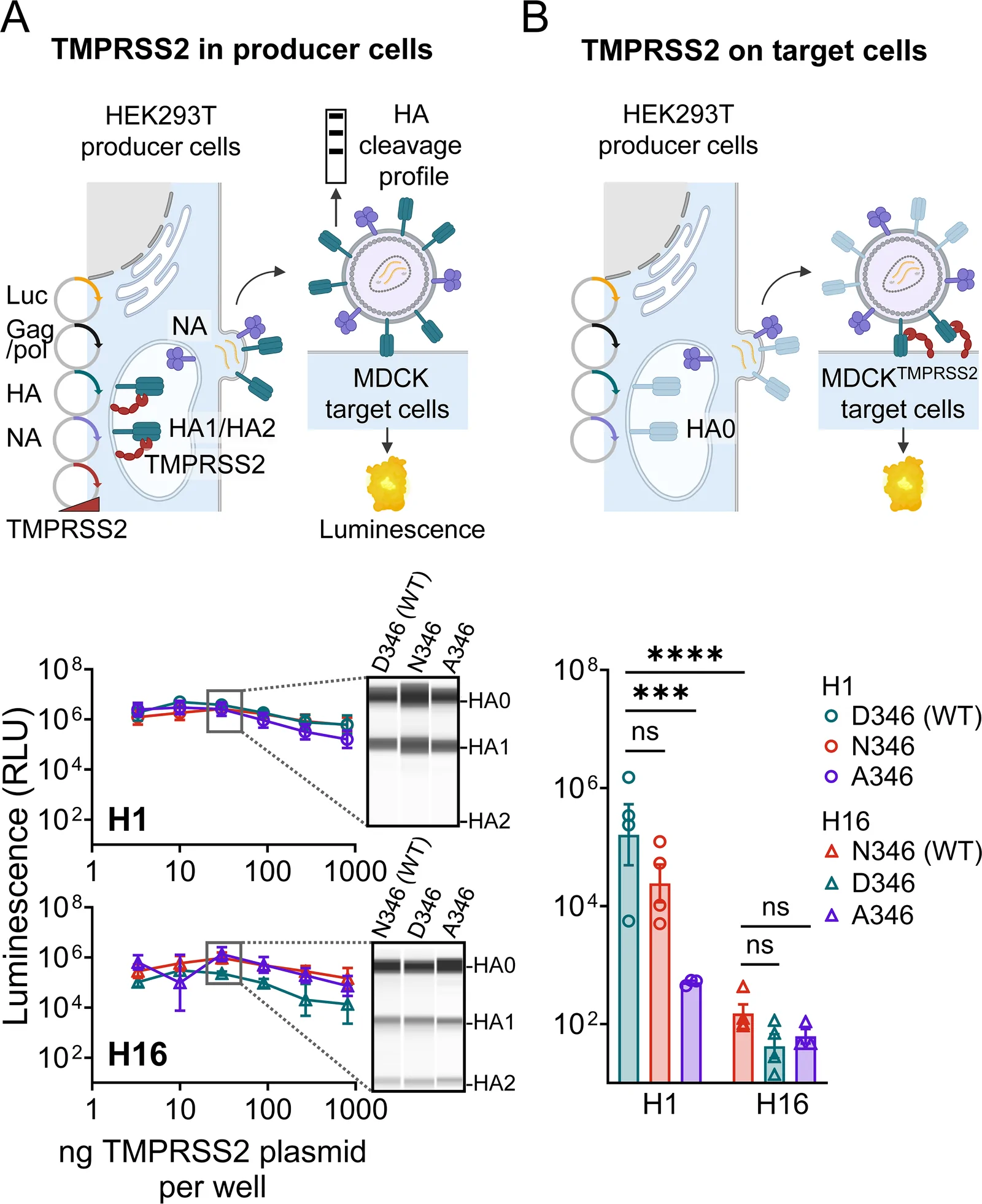
Activation of H16 HA0 by cell-associated TMPRSS2 is markedly more efficient during pseudovirus production than during target cell entry. (**A**) TMPRSS2-mediated HA0 cleavage was induced during pseudovirus production by co-transfecting a TMPRSS2 plasmid at different amounts. The activated particles were harvested and allowed to enter MDCK cells. The graphs show the mean relative luminescence unit (RLU) values ± SEM (*N* = 3), and the images show the HA0 cleavage status of pseudovirus released from the condition that received 30 ng of TMPRSS2 plasmid per well. A two-way analysis of variance (ANOVA) with Dunnett’s multiple comparison test (mutants vs WT across the different TMPRSS2 plasmid amounts) revealed no statistically significant differences. (**B**) Alternatively, the HA0-bearing pseudoviruses were harvested and exposed to TMPRSS2 during entry into MDCK^TMPRSS2^ cells. Individual and mean values ± SEM from 3 to 4 independent experiments. The differences between H1-WT vs H16-WT, H1-WT vs H1-mutants, and H16-WT vs H16-mutants were analyzed by ordinary one-way ANOVA with Dunnett’s correction for multiple comparisons. ****P ≤ 0.0001, ***P ≤ 0.001, ns: *P* > 0.05

While exogenous trypsin is commonly used to propagate IAV in cell lines that lack an HA-activating protease (e.g., MDCK cells) (28), virus activation in the human airways involves trypsin-like proteases such as TMPRSS2, among others (9, 12). TMPRSS2 is present at the cell surface, with its protease domain facing the extracellular space, but accumulates especially within the Golgi and TGN (18, 19), enabling it to cleave newly synthesized HA0 during intracellular trafficking (18, 19, 29). In human airway epithelium models, the latter (i.e., intracellular) activity was shown to be more relevant than extracellular HA0 cleavage (18), potentially due to cell surface TMPRSS2 having lower enzymatic activity (29). In this context, we assessed pseudovirus activation by TMPRSS2 in two setups (Fig. 3). In setup A, TMPRSS2 was co-expressed during pseudovirus production. Varying the amount of TMPRSS2 plasmid allowed us to modulate the expression level, as confirmed by flow cytometry (Fig. S2). In setup B, non-activated pseudoviruses were produced and subsequently transduced into MDCK^TMPRSS2^ cells, which stably express abundant TMPRSS2 at the cell surface (Fig. S2).

Somewhat surprisingly, when TMPRSS2 was present in the producer cells (Fig. 3A), we saw no entry difference for the H1N1 pseudoviruses bearing WT (H1^D346^) or mutant (H1^N346^ or H1^A346^) HA. There was also no statistically significant difference between the H16N3 pseudoviruses bearing H16^N346^ (WT), H16^D346^, or H16^A346^ HA. On the other hand, when TMPRSS2 acted at the point of viral entry (Fig. 3B), impaired entry was seen for H1N1 particles bearing H1^A346^ HA (*P* < 0.001 vs WT); we also saw a reduction for the H1^N346^ mutant, but this was not significant vs WT. Quite strikingly, we noticed that H16N3-WT (H16^N346^) entered ~1,000-fold less efficiently than H1N1-WT (H1^D346^; *P* < 0.0001). This marked difference was not observed in setup A, where both WT pseudoviruses generated luminescence signals of ~10^5^–10^6^ relative luminescence units (RLUs). Thus, while residue 346 had little to no role in either setup, the activation of H16 HA0 by TMPRSS2 was much more efficient when the enzyme was present in the producer cells compared to the setup in which TMPRSS2 was only available during target-cell entry. Compared with H1 HA0, H16 HA0 appears markedly less susceptible in the latter setting, although this difference may reflect the producer-cell context, longer TMPRSS2 exposure time during production, or both. Previous studies likewise support activation of H16 HA0 by cell-associated TMPRSS2 (10, 30).

Next, we tested setup A with plasmids encoding two other activators of HA0 (31–33). TMPRSS4 efficiently activated the H1^D346^, H1^N346^, H16^N346^, and H16^D346^ HA0s (Fig. S3), although entry levels were again significantly higher for WT-H1N1 than WT-H16N3 (*P* < 0.001). In contrast, TMPRSS13 proved to be a very poor activator of H16 HA0 (both WT and mutant). This protease efficiently activated the H1N1 particles but significantly less so (*P* < 0.05) when they carried mutant (H1^N346^) instead of WT HA (H1^D346^). Given the limited research on TMPRSS13 (such as its cleavage site preference [32]), this observation is quite difficult to interpret. It is possible that TMPRSS13 might act during virus budding, when HA0 is exposed at the plasma membrane, or extracellularly after virus release. The latter might explain the apparent parallel with exogenous trypsin activation.

To conclude, mutation D346N in H1 HA0 caused a severe cleavage defect for exogenous trypsin but had little to no effect when activation was mediated by cell-associated TMPRSS2 or TMPRSS4. We confirmed the previous finding (10, 15) that H16 HA0 is quite resistant to trypsin and showed that this is partly alleviated by mutation N346D. H16 HA0 was activated much more efficiently when TMPRSS2 was present during pseudovirus production compared with the condition in which non-activated particles encountered the protease only during entry.

### TMPRSS2 overcomes the trypsin resistance of H16 HA0 during virus rescue

The H16 HA sequence that we used originates from an H16N3 virus isolated from gulls (34). Reverse engineering of this virus proved challenging (35), most likely due to the trypsin resistance of its HA0 (10, 15). To investigate the possible contribution of residue 346, we reverse-engineered chimeric H1N1 (Virg09) and H16N3 (Gull99) viruses bearing WT or mutant HA and the internal genes of PR8. Two setups were used to expose HA to exogenous trypsin or cell-associated TMPRSS2 (Table 1). In the first setup, the eight reverse genetics plasmids were transfected in a co-culture of HEK293T and MDCK cells, followed by virus expansion in MDCK cells, both in the presence of 5 µg/mL trypsin. Only the two Virg09_PR8_ viruses were successfully rescued, with WT (H1^D346^) virus reaching approximately 100-fold higher titers than the H1^N346^ mutant (Table 1). In the second setup, a ninth plasmid encoding TMPRSS2 was included, and transfection was performed in a co-culture of HEK293T and MDCK^TMPRSS2^ cells, followed by virus expansion in MDCK^TMPRSS2^ cells. This approach enhanced virus yield for both Virg09_PR8_ viruses, particularly the H1^N346^ mutant, and was essential for rescuing the Gull99_PR8_ viruses, with the H16^D346^-mutant producing slightly higher titers than the H16^N346^ (WT) virus. Sequencing confirmed that neither of the four rescued viruses acquired additional mutations.

**TABLE 1:** Virus rescue of Virg09_PR8_ (H1N1) and Gull99_PR8_ (H16N3) in MDCK and MDCK^TMPRSS2^ cells

Virus and HA mutation	Rescue^*a*^ in co-suspension of HEK293T and	
	MDCK cells + trypsin	MDCK^TMPRSS2^ cells + TMPRSS2 plasmid
	Virus titer (log_10_ CCID_50_/mL) in MDCK^*b*^ No. positives/total^*c*^	
Virg09_PR8_ (H1N1)—H1^D346^ (WT)	6.3 (3/3)	7.5 (2/2)
Virg09_PR8_ (H1N1)—H1^N346^	4.3 (3/3)	6.8 (2/2)
Gull99_PR8_ (H16N3)—H16^N346^ (WT)	ND (0/3)	2.0 (2/3)
Gull99_PR8_ (H16N3)—H16^D346^	ND (0/3)	2.9 (2/3)

^*a*^ The reverse genetics plasmids were transfected in a co-suspension of HEK293T cells and (left column) MDCK cells, in medium with 5 µg/mL trypsin; or (right column) MDCK^TMPRSS2^ cells and co-transfected with a TMPRSS2 plasmid.

^*b*^ The supernatants were titrated in MDCK cells receiving 5 µg/mL trypsin. The values shown are the average CCID_50_ titers of 2 or 3 independent rescues. ND: not detectable, i.e., titer below 2 log_10_ CCID_50_/mL.

^*c*^ Between brackets: number of successful rescues over total.

Growth kinetics further supported these findings. In trypsin-supplemented MDCK cells, the Virg09_PR8_ virus bearing mutant HA (H1^N346^) replicated more slowly than the WT (H1^D346^; *P* < 0.001 at 24 h post-infection (p.i.); Fig. S4), whereas in MDCK^TMPRSS2^ cells, no significant difference was noted. For the Gull99_PR8_ virus assessed in trypsin-supplemented MDCK cells, the H16^N346^ (WT) form showed somewhat faster growth than the H16^D346^-mutant (*P* < 0.01 at 24 h p.i.; Fig. S4). However, sequencing of progeny viruses revealed that the Gull99_PR8_-WT virus acquired substitution N346D by 72 h p.i. in both MDCK and MDCK^TMPRSS2^ cells (Fig. S4; sequencing result shown at 72 h p.i.). Analysis of the initial virus stock showed that a minor subpopulation had already acquired this mutation during virus expansion (Fig. S4; sequencing result shown at 0 h p.i.). All this supports that HA residue D346 is strongly favored over N346 for efficient IAV replication in MDCK cells.

### Besides its poor activation by exogenous trypsin, H16 HA is atypical in having an unusually low fusion pH

**Fig 4:**
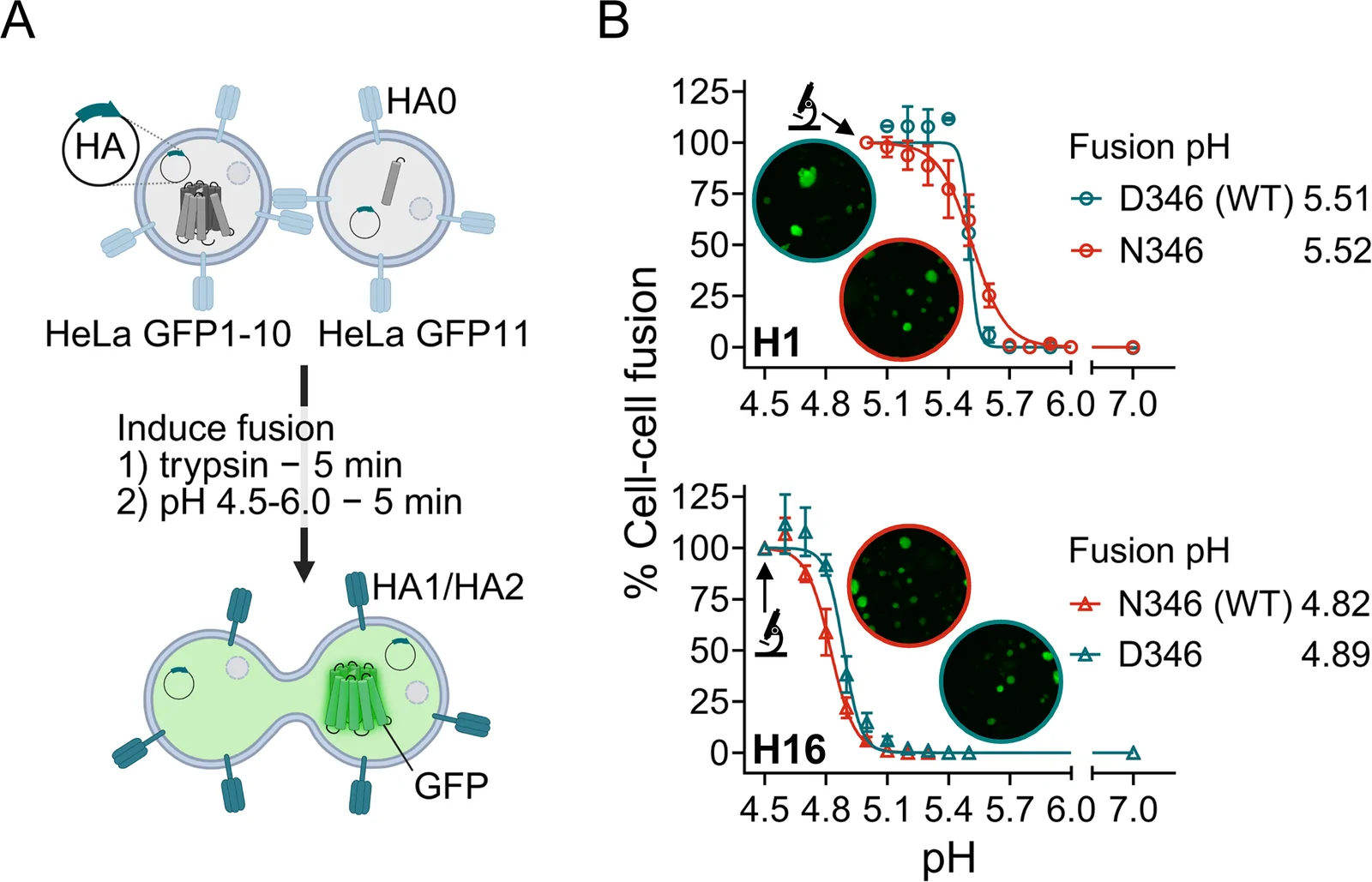
Fusion pH of D346N-mutant H1 HA and the reverse mutant of H16 HA. (**A**) Scheme of the split-GFP cell-cell fusion assay in HA-transfected cells exposed to low pH. (**B**) pH profiles of H1^D346^ (WT) and H1^N346^ HA (top); and H16^N346^ (WT) and H16^D346^ HA (bottom). The Y-axis shows the percent cell-cell fusion, calculated by dividing the GFP area (after background subtraction) at each pH by the one at pH 5.0 (H1) or pH 4.5 (H16). The legend shows the fusion pH, defined as the pH where fusion was 50% relative to pH 5.0 (H1) or pH 4.5 (H16). Data points are the mean ± SEM (*N* = 3). The insets show representative images of the green fluorescence in the condition exposed to pH 5.0 (H1) or pH 4.5 (H16) of each WT and mutant.

Knowing that a high concentration of trypsin was required to activate H1^N346^-mutant HA, we applied this condition to determine the fusion pH by split-GFP cell-cell fusion assay (Fig. 4A). A co-suspension of the two cell lines was transfected with the HA plasmid, and 2 days later, the monolayers were treated with trypsin and exposed to a range of acidic buffers. After 24 h, green fluorescent syncytia were quantified by high-content imaging.

The results (Fig. 4B) show that the fusion pH was nearly identical for the H1^D346^ and H1^N346^ HAs (5.51 vs 5.52), as well as for the H16^N346^ and H16^D346^ proteins (4.82 vs 4.89). The value for WT Virg09-HA (H1^D346^) aligns with our previous study (33) and the value of 5.5 for another early A(H1N1)pdm09 isolate (14). The most striking observation was the unusually low fusion pH of 4.82 for H16^N346^ (WT) HA. Since this sequence is derived from a gull H16N3 isolate, we had expected a higher fusion pH in the range of 5.6–6.0, which is typically seen for avian HAs (36).

Hence, besides its resistance to exogenous trypsin, H16 HA has a second peculiarity in showing an exceptionally low fusion pH of 4.8, far below the values of other avian HAs. This might reflect a host-specific adaptation, as H16N3 viruses were so far mainly detected in gull species (37).

### Four other identified mutations render H1 HA more acid stable

**Fig 5:**
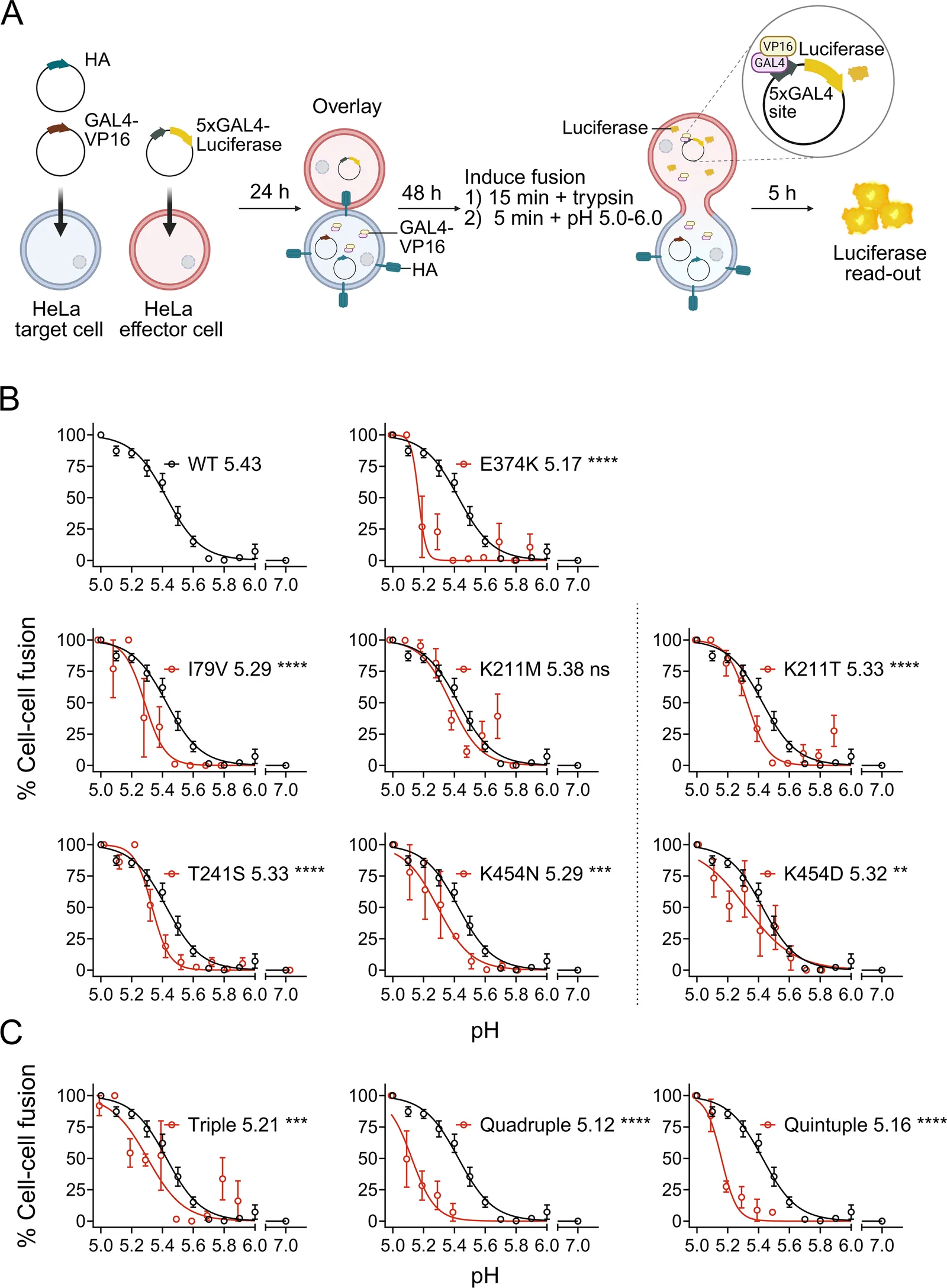
pH profile of the mutations that render H1 HA more acid-stable. (**A**) Scheme of the luciferase reporter-based cell-cell fusion assay in HA-expressing cells exposed to low pH. (**B and C**) pH profiles for WT HA (black curve shown in each graph) and mutant forms of HA (curves in red). (**B**) WT, reference mutation E374K, four single mutants that exhibited a shift in fusion pH, and two variations. (**C**) Triple (I79V + K211T + K454N), quadruple (triple + E374K), and quintuple mutant (quadruple + T241S). In each graph, the Y-axis shows the percent cell-cell fusion, calculated by dividing the luminescence signal (after background subtraction) at each pH by the one at pH 5.0. The legend shows the fusion pH, defined as the pH where fusion was 50% relative to pH 5.0. Statistical significance is shown for the difference between mutant and WT (extra sum of squares *F*-test of best-fit value). Data points are the mean ± SEM (*N* = 3–4). ****P ≤ 0.0001, ***P ≤ 0.001, **P ≤ 0.01, ns: *P* > 0.05

In the next part, we focused on the other nine mutations that we identified after incubating mutant libraries of Virg09_PR8_ virus at pH 5.0 (see the first part of the results). Using a luciferase-based cell-cell fusion assay (Fig. 5A), we measured a fusion pH of 5.43 for WT Virg09-HA (Fig. 5B). Within 3 years after the 2009 pandemic, all isolates carried the stabilizing HA mutation E374K (E47_2_K in H3-numbering; Table S1) (22, 24). For this reference mutation, we measured a fusion pH of 5.17 (Fig. 5B), which is 0.26 pH units lower than the WT (*P* < 0.0001). Among the mutations that we identified, three changes (i.e., I79V, T241S, and K454N) caused a significant increase in acid stability (*P* < 0.001 or *P* < 0.0001 vs WT). Mutation K211M caused a non-significant decrease of 0.05 pH units, but engineering the K211T mutant instead reduced the fusion pH by 0.11 units (*P* < 0.0001 vs WT). Five of the nine HA mutations had no significant or an opposite (i.e., higher fusion pH) effect on acid stability (Fig. S5) and were therefore omitted from further study.

Since the stabilizing mutations are located at distinct regions in HA (see last section of results), we wondered whether combining them might yield a cumulative stabilizing effect. Therefore, we evaluated HAs bearing I79V + K211T + K454N (triple mutant; fusion pH 5.21), combined with E374K (quadruple mutant; fusion pH 5.12), and T241S (quintuple mutant; fusion pH 5.16; Fig. 5C). Hence, the triple mutant was only slightly more stable than the single I79V, K211T, and K454N mutants (fusion pH: 5.29, 5.33, and 5.29, respectively; Fig. 5B). Similarly, the quadruple and quintuple mutants barely reached higher stability than E374K alone (fusion pH: 5.17). Thus, the combined effect of the mutations is, at best, modestly additive, confirming previous findings that the combination of stabilizing mutations does not commonly enhance the overall stability of HA (38).

### The stabilizing HA mutations cause subtle impairment of HA fusion activity and of viral entry and replication in human airway-derived cells

**Fig 6:**
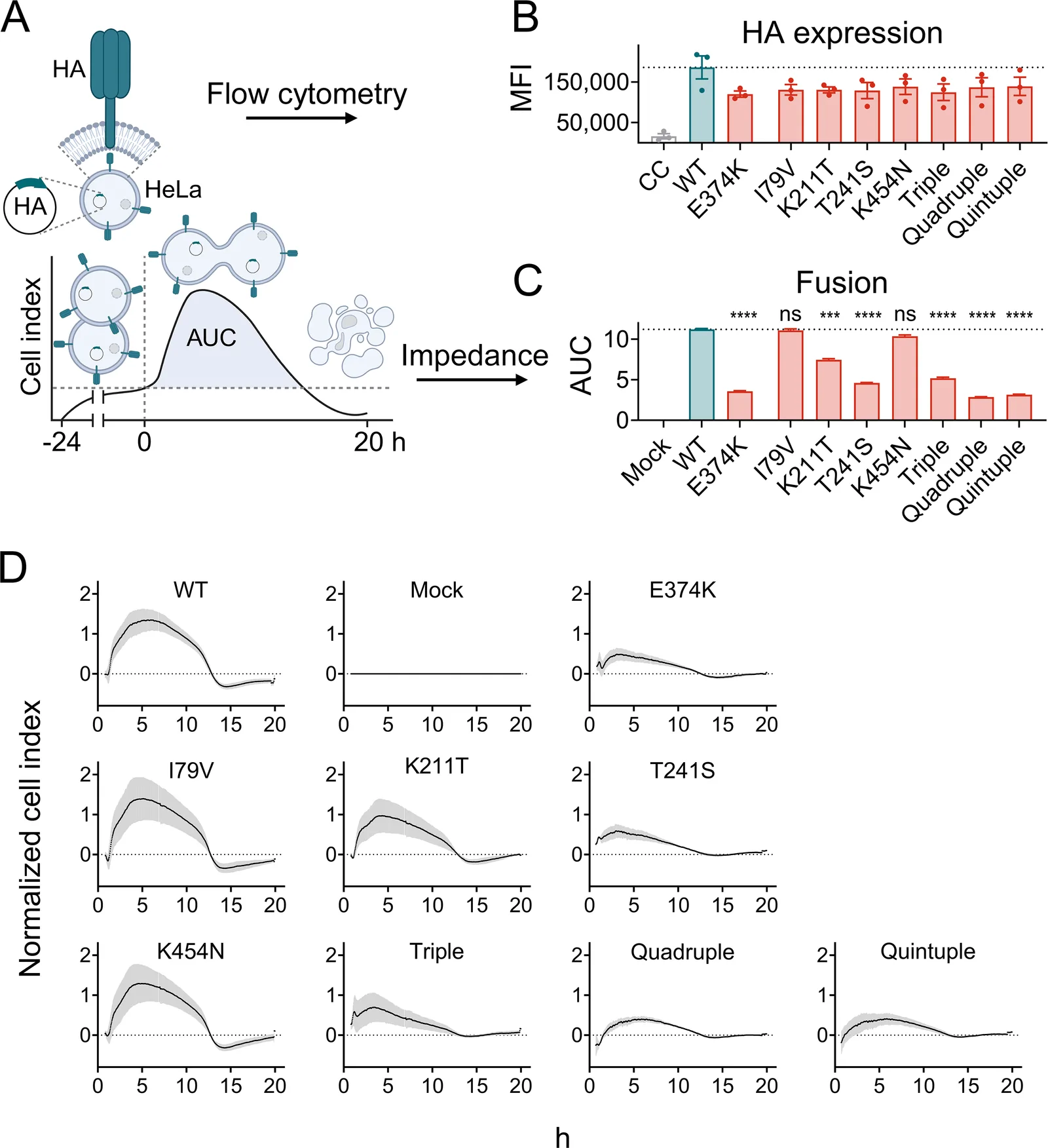
The acid-stabilizing mutations in H1 HA affect the efficiency of cell-cell fusion. (**A**) Scheme of the impedance assay. (**B**) HeLa cells were transfected with WT or mutant HA and, 48 h later, analyzed for HA surface expression using flow cytometry. CC: cell control transfected with empty plasmid. MFI: mean fluorescence intensity; individual and mean values ± SEM (*N* = 3). For neither of the mutants, the MFI was significantly different compared to the WT (ordinary one-way analysis of variance [ANOVA] with Dunnett’s corrections for multiple comparisons). (**C and D**) In parallel, the cells were sequentially exposed to trypsin and pH 5.0 (time 0) to induce syncytium formation, which was followed by measuring the impedance during 20 h. (**C**) Area under the curve (AUC) for each HA was calculated from the pooled cell index data from three (mutants) or six (WT, mock, and E374K reference) independent experiments. For each mutant except for I79V and K454N, the difference vs WT was significant (Brown-Forsythe and Welch ANOVA, followed by post-hoc comparisons using Dunnett’s T3 test). Mock: cells transfected with an empty (instead of HA) plasmid. (**D**) Plots showing the normalized cell index as a function of time. The dotted lines indicate the baseline that was used to normalize the values, which was measured right before cell-cell fusion was induced. ****P ≤ 0.0001, ***P ≤ 0.001, ns: *P* > 0.05

To assess the impact of the stabilizing mutations on the fusion activity of HA, we used a real-time impedance assay, modified from our previous method for the coronavirus spike protein (39) (Fig. 6A). HA-expressing HeLa cells were cultured until confluency, resulting in a gradual increase in cell index (CI). Upon treatment with trypsin followed by pH 5.0, cell-cell borders dissolved and syncytia formed, giving an increase in electrical resistance and a sharp rise in CI. This was followed by a decline, due to syncytial instability and cell lysis. Fusion efficiency was quantified as the area under the curve (AUC), which correlates with syncytium size (39).

With the exception of I79V and K454N, all mutants showed significantly lower AUC values than the WT (*P* < 0.0001 or *P* < 0.001, Fig. 6C). E374K caused the most pronounced reduction (3.1-fold) in syncytium size, while K211T and T241S led to moderate reductions (1.5- and 2.4-fold, respectively). Syncytium formation was further impaired for the triple, quadruple, and quintuple mutants (Fig. 6C and D). The impaired fusion activity was not explained by a difference in HA surface expression, since these levels proved comparable for all mutants (see flow cytometric analysis in Fig. 6B).

To assess viral entry, H1N1 pseudoviruses were produced in HEK293T cells and activated with trypsin. After transduction in MDCK and A549 cells, luciferase activity was measured 72 h later (Fig. S6, panel A). In MDCK cells, all mutants generated signals comparable to WT (~10^6^ RLU; not significant for all single mutants except for E374K [*P* < 0.05]). In human lung-derived A549 cells, the differences were more pronounced, the impairment being significant (*P* < 0.001) for the I79V and K211T mutants, and even more so (*P* < 0.0001) for the E374K, quadruple, and quintuple mutants (Fig. S6, panel B).

**Fig 7:**
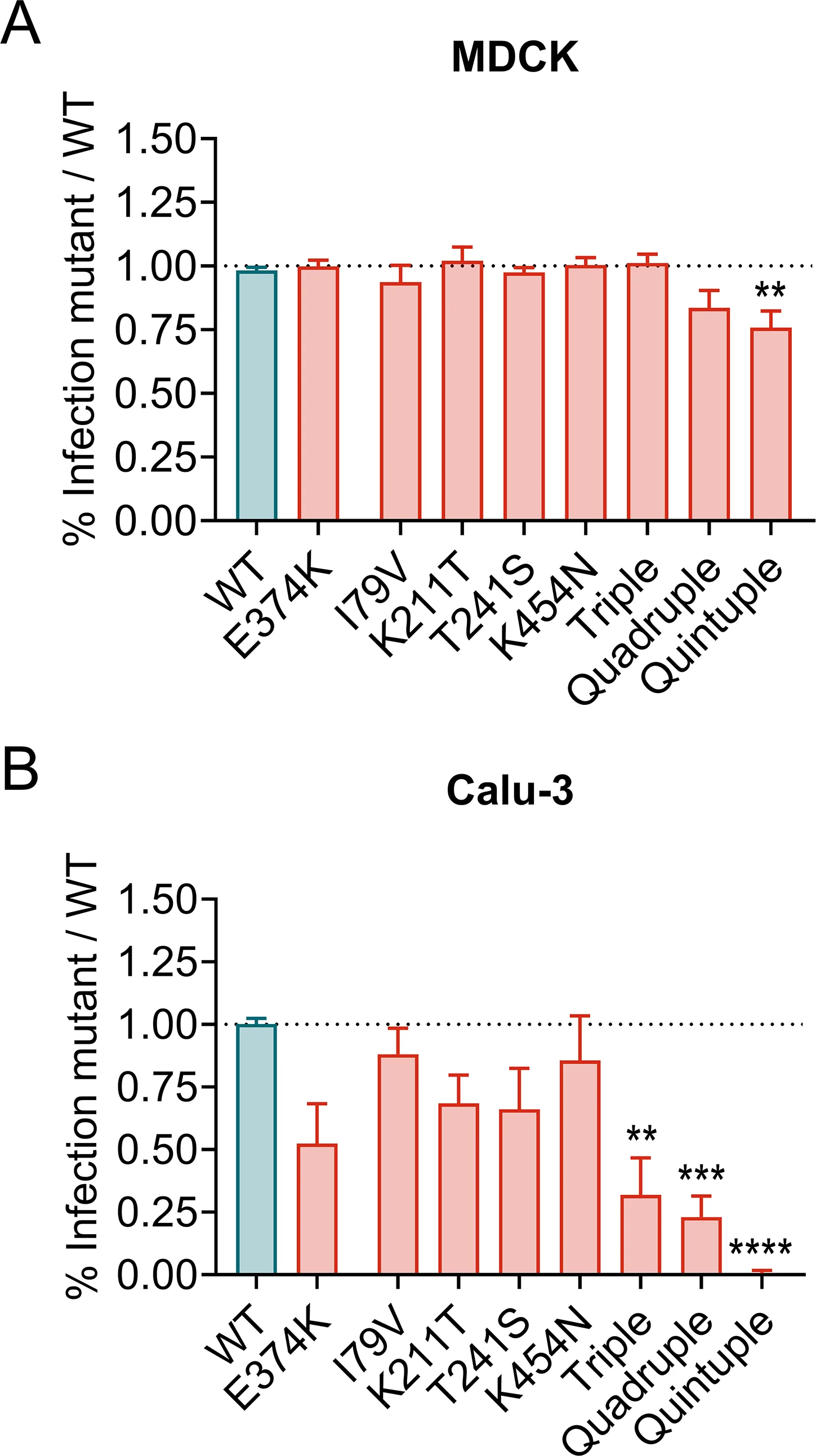
Replication efficiency of HA-mutant Virg09_PR8_ viruses in MDCK and Calu-3 cells. MDCK cells (**A**) or Calu-3 cells (**B**) were infected with reverse-engineered Virg09_PR8_ viruses. At day 3 p.i., the percent infected cells was quantified by immunostaining and high-content imaging. Data are expressed as percent infection relative to WT and shown as individual and mean values ± SEM from three independent experiments. Differences between mutants and WT were analyzed by ordinary one-way analysis of variance (ANOVA) with Dunnett’s correction for multiple comparisons. ****P ≤ 0.0001, ***P ≤ 0.001, **P ≤ 0.01, ns: *P* > 0.05

Finally, reverse-engineered Virg09_PR8_ viruses carrying the single or combined HA mutations replicated to high titers (Table S2) and retained their mutation(s) after four passages in MDCK cells, indicating little to no impairment of viral fitness. In some viruses, one to three additional HA mutations emerged, which might have been compensatory, as their prevalence declined after low-pH selection (Table S3). We also assessed the WT and mutant Virg09_PR8_ viruses for multicycle replication in MDCK and Calu-3 cells. While little to no differences were seen in MDCK cells (Fig. 7A), several acid-stable mutants, and especially those carrying three or more mutations, exhibited impaired replication in human airway-derived Calu-3 cells (Fig. 7B).

To summarize, our random mutagenesis and pH 5.0 selection approach yielded four sites in H1 HA (I79, K211, T241, and K454), which were not previously implicated in HA acid stability. Mutations at these sites reduce the fusion pH and fusion activity of HA and subtly impair the efficiency of viral entry and replication in human airway-derived cell lines.

### Most of the mutated residues show a high conservation rate in H1 HA

**Fig 8:**
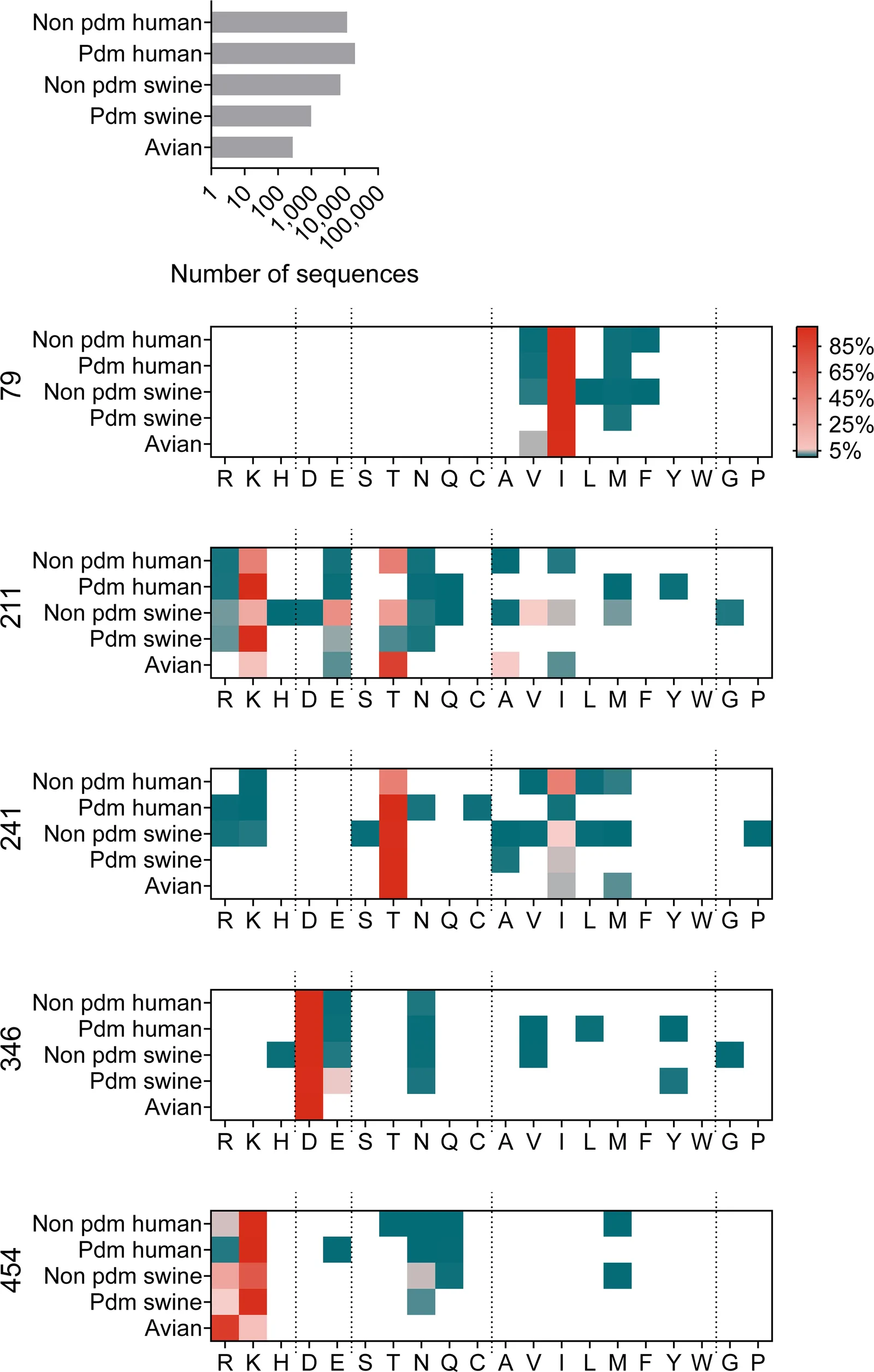
Conservation rate of the acid-stability determining residues in different subgroups of H1 HA. The Influenza Research Database online single-nucleotide polymorphisms tool was used to determine the percentage of sequences bearing any of the 20 amino acid residues at the five mutated positions. From left to right and separated by a dotted line: basic, acidic, neutral, hydrophobic, and other residues. Note that the number of sequences was not equal in the five subgroups (see graph at the top). The heatmaps visualize the percentage of the sequences bearing the residues on the X-axis, with the color legend shown on the right.

Using the single-nucleotide polymorphisms tool in the Influenza Research Database (40), we assessed the natural variation of the five sites in human, swine (pandemic or non-pandemic), or avian H1 sequences. The results are shown as a heatmap in Fig. 8.

Overall, four of the five residues showed fairly high conservation in H1 HA. Substitutions of R/K454 are very rare, with almost zero occurrence of a negatively charged residue. Based on this, we created K454D-mutant Virg09 HA for testing in the cell-cell fusion assay. Its fusion pH was 5.32 (Fig. 5B), which was significantly lower than the value of WT (*P* < 0.01) and similar to that of mutant K454N (fusion pH 5.29).

The fifth residue, K211, appeared to be the least conserved and tolerant to different amino acids. While variation M211 proved rarely present, T211 is very common and even dominant in the avian reservoir. In non-pandemic swine strains, E211 is quite widespread.

Hence, except for K211T, the stabilizing mutations identified in our study involve residues that are highly conserved among circulating H1 HAs, even across human, swine, and avian hosts.

### Interpretation of the biological data in relation to the HA protein structure

**Fig 9:**
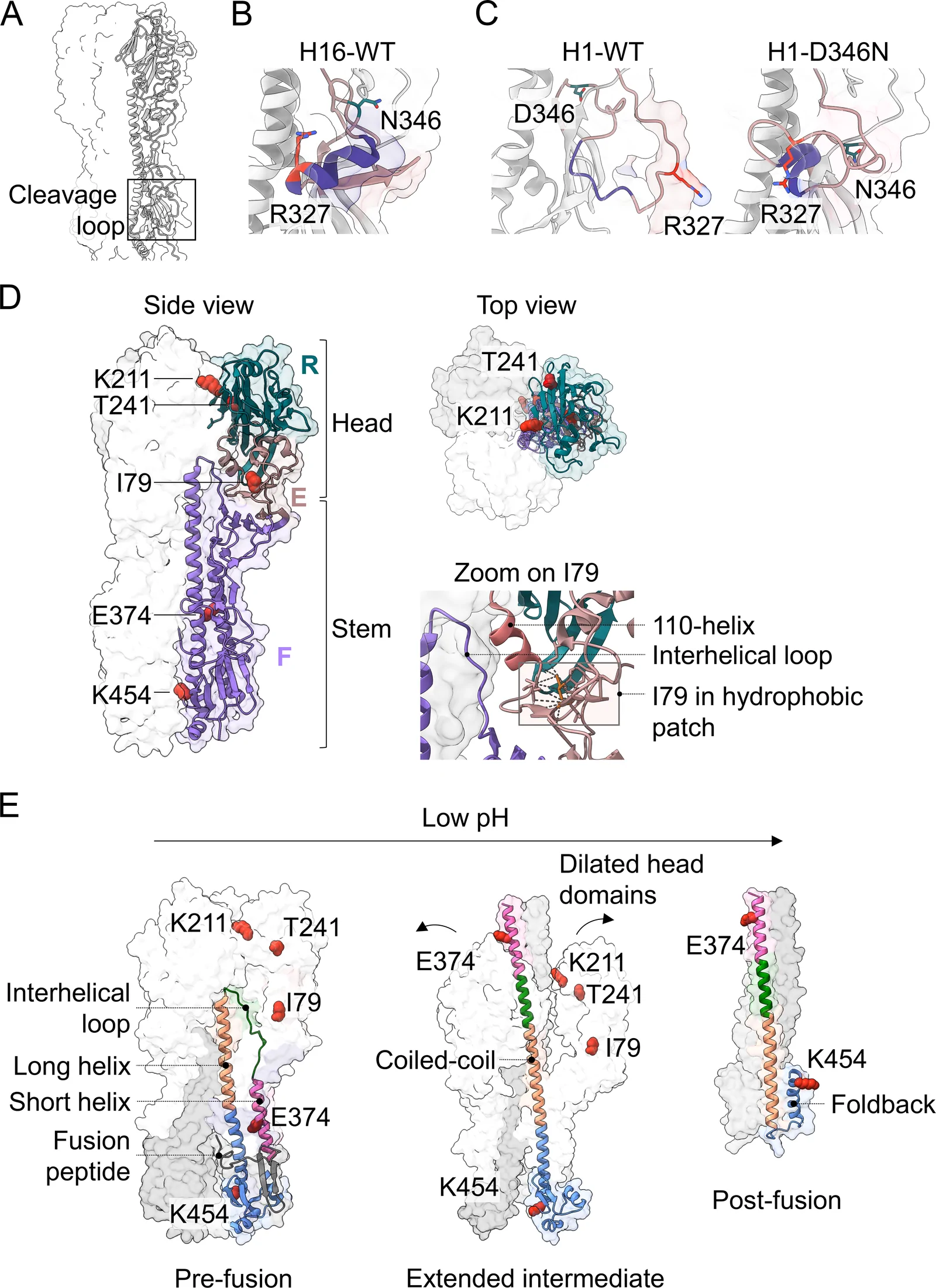
Location of the cleavability- and stability-determining residues in the HA protein structure. (**A**) Visualization of the HA0 precursor protein with the location of the cleavage loop marked by a black box. (**B**) In the X-ray structure of the H16 HA0 cleavage loop (PDB 4F23) (15), the scissile R327 residue (in red) is hidden behind a short α-helix (in purple). Residue N346 is shown in teal. (**C**) We created AlphaFold models of H1^D346^ (WT) and H1^N346^ HA0 proteins (45). This residue change (in teal) is predicted to induce an extra turn (in purple) in the cleavage loop, which may reduce the access of R327 (in red) to proteases. (**D**) Left panel: HA trimer in pre-fusion conformation, with one protomer colored according to receptor (R; in teal), vestigial esterase (E; in beige), and fusion (F; in purple) subdomain. The red spheres indicate the location of the five stabilizing mutations that we studied. Right panel, upper figure: top view visualizing the location of K211 and T241 at an inter-protomer interface. Right panel, lower figure: I79 lies in a hydrophobic patch adjacent to the 110-helix of the E subdomain, which interacts with the interhelical loop of the F subdomain. (**E**) During the low pH-induced transition from the pre- to post-fusion structure, there is dilation of the three head domains. After formation of the extended coiled-coil (shown in pink, green, orange, and blue in the extended intermediate), the C-terminal part (colored in blue with K454 shown as a red sphere) folds back by 180°, resulting in the final post-fusion structure. Models generated from PDB 3M6S (46) (with a few residues altered to fit Virg09), PDB 6Y5K (5), and PDB 1HTM (47) (using SWISSMODEL to map the Virg09 sequence onto the structures).

In the final part, we sought how the biological behavior of the mutant HAs could be explained from the protein structure. For mutation D346N, we analyzed the folding of the cleavage loop (Fig. 9A), which shows prominent differences in the few HA0 structures that are so far available (15, 41–44). A striking feature in the cleavage loop of H16 HA0 (PDB 4F23, 15) is the presence of a short α-helix, which positions scissile residue R327 deep in the stem domain (Fig. 9B) and likely contributes to the trypsin resistance of this subtype (15). Interestingly, AlphaFold models of the Virg09 HA0 cleavage loop, in H1^D346^ (WT) or H1^N346^-mutant form, predicted distinct conformations near the mutation site (Fig. 9C). Whereas the WT model shows a more solvent-exposed loop, the mutant model carries an additional turn, which buries the loop deeper into the HA stem. Consequently, the scissile R327 residue is accessible in the WT (H1^D346^) HA0 protein but less so in the mutant (H1^N346^), offering a structural explanation for the observed cleavage defect.

To interpret the acid-stabilizing mutations that we identified, we mapped the mutated residues onto the crystal structure of prefusion A/Darwin/2001/2009 H1 HA (PDB 3M6S [46]), obtained at neutral pH, and onto structures of the post-fusion and intermediate states, PDB 6Y5K (5) and PDB 1HTM (47). Our mutations are situated in the globular head as well as the stem domain (Fig. 9D), consistent with the common insight that stabilizing mutations can occur throughout the entire HA (26, 27, 48).

During refolding of the HA trimer at acidic pH, the three membrane-distal domains separate via dilation and rotation (5). This offers a possible explanation for the stabilizing effect of mutations K211M and T241S, which are located at an inter-protomer interface in the R subdomain (Fig. 9D; top view on the right). Residue I79 is located in the E subdomain, which connects the R and F subdomains. Intriguingly, it is located in a hydrophobic patch adjacent to the 110-helix of the R subdomain (Fig. 9D; bottom right). This helix interacts with the interhelical loop of the F subdomain, which undergoes a loop-to-helix transition at low pH. Given that mutations in the 110-helix have been shown to affect acid-stability of H5 HA (49), the same seems valid for mutations in the nearby hydrophobic patch. The I79V mutation retains sidechain hydrophobicity but slightly reduces its size.

After the interhelical loop of the F subdomain transitions into a helix, a long coiled-coil structure is formed that extends toward the head domain (Fig. 9E). Then, the C-terminal part of the long helix folds back by 180°, causing its relocation upwards and alongside the coiled-coil core of the final post-fusion structure. Mutation K454N, situated in this fold-back region, causes a loss of net charge, besides reducing the size and flexibility of the sidechain. Finally, the known stabilizing mutation E374K, which we included as a reference, is located in the short helix of the F subdomain and introduces a salt bridge between K374 and E21 of the adjacent monomer, thereby enhancing H1 HA trimer stability (22, 24).

## DISCUSSION

The influenza virus HA is subject to structural instability, antigenic drift, and host adaptation. Understanding these processes at the level of specific HA regions or residues remains a key area of interest. In this study, we employed a random mutagenesis approach, which, though more limited than previous mutagenesis studies conducted on H5 HA (50, 51) or H3 HA (52), enabled us to identify mutations that increase the acid stability of H1 HA and advance the growing knowledge in this field (13, 26, 27).

Though not our starting objective, we serendipitously discovered a role for residue D346 in modulating HA0 cleavage. A mutant H1N1 pseudovirus bearing N346 required 12-fold more trypsin than the WT (D346), and, reciprocally, replacing N346 with D346 reduced the trypsin requirement of H16N3 pseudovirus by approximately fourfold. According to our structural model (which awaits experimental validation), the presence of N346 alters the conformation of the cleavage loop, reducing the accessibility to trypsin. On the other hand, TMPRSS2 proved an efficient activator of the N346-bearing H1 and H16 proteins (the latter was reported before [10, 30]), particularly during pseudovirus production. This aligns with reports that the cleavage of H1 HA0 by TMPRSS2 can occur during intracellular trafficking and may be more relevant than cell-surface cleavage (18, 29). However, our assay did not distinguish whether cleavage in pseudovirus production occurred intracellularly during HA trafficking, at the producer-cell surface, or through a combination of both. While both H1HA0 and H16 HA0 were efficiently activated when TMPRSS2 was present during pseudovirus production, the difference in target-cell entry was much more pronounced for H16 HA0. This might suggest that, especially for H16 HA0, the shape and accessibility of the cleavage loop profoundly change during HA biosynthesis, glycosylation, trimerization, trafficking, or viral budding and release (53). It is plausible that the short α-helix shielding the scissile arginine in H16 HA0 (15) only forms at a later stage. Alternatively, upon HA/TMPRSS2 co-expression, side-by-side contact between the two proteins presented at the plasma membrane might trigger HA0 cleavage at the cell surface. This might not be possible when virion-associated HA0 forms head-on-head contact with TMPRSS2 that is exposed on the surface of the target cell. Looking at the cleavage motif in the two HA0 proteins (Fig. 2A: IQSR↓ in H1 and INER↓ in H16), the H16 protein contains residues at P2 (E) and P3 (N) that might be less favorable than those in H1 (P2: S and P3: Q), for cleavage by TMPRSS2 (16). For trypsin, both motifs might be recognized to a similar extent (17). Hence, it seems relevant to perform experiments in which the exogenous trypsin, used in our study, is replaced by soluble recombinant TMPRSS2; however, the suitability of commercial forms of this protease appears variable (54). Anyway, the requirement for activation in the producer-cell context also explains why co-expression of TMPRSS2 proved essential to reverse-engineer the H16N3 (i.e., Gull99_PR8_) virus. In a previous report on the unsuccessful rescue of this virus in trypsin-supplemented cells, the problem was solved by inoculating the transfected cells into embryonated eggs (35). In our experiments, trypsin and TMPRSS2 served as model proteases. So far, the identity of the proteases responsible for activating H16N3 virus in its natural host, gulls, remains unknown.

Our findings might indicate a potential role of the gull H16N3 virus in influenza virus zoonosis (55, 56). First, the low fusion pH (~4.8) of H16 HA resembles that of human IAVs, which typically have lower fusion pH values than avian strains (14, 36). Combined with its ability to bind α2,6-sialylated glycans (57) and its susceptibility to human TMPRSS2 in virus-producing cells (this study and references 10, 30), the gull H16N3 virus appears well adapted for attachment to the human respiratory tract (58) and for replication within this tissue. Second, its high acid-stability and resistance to cleavage by extracellular proteases may enhance environmental persistence. Gulls inhabit coastal areas where the high salinity of seawater can reduce viral persistence (59) and promote inactivation in evaporating droplets (60). The H16N3 virus appears well adapted to survive under these harsh circumstances.

In the second part of this study, we initially recovered 10 H1 HA mutations at 9 different sites after random mutagenesis and low pH selection, of which 4 sites (i.e., I79, K211, T241, and K454) proved amenable to stabilizing mutations. The other selected mutations may reflect subtle or context-dependent effects that conferred an advantage during the combined selection-and-expansion procedure but were not captured in the fusion pH assay. We confirm the common knowledge (26, 27, 48) that mutations in the globular head, vestigial esterase, and membrane-proximal part can have a profound impact on HA acid stability, particularly when located at inter-monomer interfaces or regions critical for low-pH-induced structural transitions. We saw that combining multiple stabilizing mutations was, at best, modestly additive, which aligns with a previous report (38). The authors proposed a hierarchical influence, where substitutions in the short α-helix of the stem domain (E374K in our study) may override those in the membrane-distal region (here K454N), and substitutions near the fusion peptide may dominate over those in the other two regions (here K211T, T241S, and I79V). Anyway, the possibility that stabilizing mutations can occur in diverse regions of the protein complicates the interpretation of HA sequence changes during zoonotic IAV surveillance.

In retrospect, using more stringent selection conditions (e.g., lower pH or longer incubation) might have yielded mutants with even greater acid-stability. However, an inactivation pH of 4.9–5.0 seems to be the lower threshold for replication-competent viruses, with only a few strains exhibiting such prominent stability (13, 36). While engineered mutations can yield HA proteins that are stable at pH 4.7 (61) or even 4.3 (62), such extreme stabilization likely impairs viral replication. After endocytosis, influenza virions encounter progressively acidic environments: early endosomes (pH 6.5–6.0), late endosomes (pH 5.5–5.0), and lysosomes (pH 4.5–5.0; 63–65). A virus with a very low fusion pH may fail to fuse before reaching lysosomes, where it faces interferon-inducible factors (66). The very low fusion pH of ~4.8 that we measured for Gull99-H16 HA is quite intriguing and reminiscent of the low HA activation pH (i.e., 5.0) reported for an avian H1N1 virus from shorebirds (67). To our knowledge, this is the first report highlighting the distinctively low fusion pH of H16 HA. Previous studies either failed to determine its fusion pH, likely due to insufficient trypsin activation (10), or reported a much higher value of 5.5 using a syncytium assay with live Gull99 H16N3 virus and Vero cells (36). Follow-up studies may help to resolve this discrepancy.

Our study also addressed whether altered HA fusion properties have an impact on viral replication fitness. Besides rendering H1 HA more acid stable, three out of five HA mutations that we studied impaired cell-cell fusion efficiency, as evidenced by smaller syncytia in our impedance assay. Next, pseudovirus entry was hardly reduced in MDCK cells but was clearly more affected in A549 cells. This could be explained by the low endosomal pH of MDCK cells, as measured by Murakami et al. (68). In terms of virus replication, the reverse-engineered acid-stable mutants reached titers comparable to WT and retained their mutations when passaged in MDCK cells. On the other hand, we did observe impaired replication in human airway-derived Calu-3 cells. This cell-type difference may reflect a more permissive entry environment in MDCK cells, possibly due to a lower endosomal pH than in Calu-3 cells. In addition, Calu-3 cells may mount a stronger interferon response (69), which could further disadvantage acid-stable mutants, particularly when delayed fusion causes trafficking toward the late endosomes. The smooth replication of HA-stabilized viruses in MDCK cells is an advantage for vaccine production, where mutations at key sites in the HA trimer may help to produce vaccines with superior shelf-life, without compromising viral yield (24).

To conclude, our random mutagenesis and pH-based selection approach revealed four stabilizing mutations in H1 HA, located across multiple domains. This confirms the complex interplay between all parts of HA during low pH-induced refolding. A fifth mutation, D346N, drastically reduced trypsin-mediated cleavage but had no effect on activation by cell-associated TMPRSS2. The presence of N346 in gull-derived H16 HA, which we found to exhibit an unusually low fusion pH, contributes to its trypsin resistance. Together, our results contribute to understanding the determinants of HA cleavability and stability, with implications for IAV surveillance and vaccine production.

## MATERIALS AND METHODS

### Cells and media

MDCK cells were provided by M. Matrosovich (Marburg, Germany). MDCK^TMPRSS2^ cells, a gift from J. Bloom (Seattle, USA), are a stable TMPRSS2-tranfectant line that was generated (70) from MDCK-SIAT1 cells, which overexpress 2,6-sialyltransferase (71). Both cell lines were maintained in Dulbecco’s modified Eagle’s medium supplemented with 10% fetal calf serum (FCS), 1 mM sodium pyruvate, and 750 mg/L sodium bicarbonate. A similar medium was used to grow HEK293T (Thermo Fisher Scientific #HCL4517), HeLa cells (ATCC #CCL-2), and A549 (ATCC #CCL-185) cells. Calu-3 (ATCC #HTB-55) cells were cultivated in Minimum Essential Medium Eagle supplemented with 10% FCS, 0.1 mM non-essential amino acids, 2 mM L-glutamine, and 10 mM HEPES. To obtain the two HeLa-based split-GFP cell lines (72, 73), lentiviruses were produced by transfecting HEK293T cells with 10 µg of the pCL-10A1 retrovirus packaging vector (Novus Biologicals), together with 5 µg of either pQCXIP-GFP1-10 or pQCXIP-BSR-GFP11 plasmid (received from Y. Hata through Addgene plasmids # 68715 and #68716), using TransIT-LT1 Transfection Reagent (Mirus Bio). Lentiviruses were harvested at 48 and 72 h post-transfection. Next, HeLa cells were seeded at 500,000 cells per well in six-well plates and transduced with 600 µL lentivirus together with 10 µg of polybrene, followed by puromycin selection 1 day later. The resulting polyclonal HeLa-GFP1-10 and HeLa-GFP11 cells were cultured in medium supplemented with 0.6 µg/mL puromycin.

### Plasmids

For reverse genetics of A/Puerto Rico/8/34 (PR8)-based chimeric IAVs, we used the pVP-PB2, -PB1, -PA, -NP, -M, and -NS plasmids encoding the internal genes of PR8, which were kindly provided by M. Kim (Korea Research Institute of Chemical Technology). In the pVP-HA plasmid, the ectodomain of the PR8 sequence was replaced by that of A/Virginia/ATCC3/2009 (A(H1N1)pdm09) (ATCC VR-1738; abbreviated Virg09), in a similar way as reported (74). In the pVP-NA plasmid, we replaced the entire NA-coding sequence of PR8 with that of Virg09. The H16 HA and N3 NA sequences of A/black-headed gull/Sweden/5/1999 (abbreviated Gull99) were ordered as GeneArt DNA strings from Life Technologies. To replace the sequences, high-fidelity PCR (Platinum SuperFi PCR, Invitrogen) was performed on the PR8 pVP-plasmids, and Virg09-HA and -NA plasmids prepared earlier (33), using overlapping primers. For reverse genetics of A/Puerto Rico/8/34 (PR8)-based chimeric IAVs, we used the pVP-PB2, -PB1, -PA, -NP, -M, and -NS plasmids encoding the internal genes of PR8, which were kindly provided by M. Kim (Korea Research Institute of Chemical Technology). In the pVP-HA plasmid, the ectodomain of the PR8 sequence was replaced by that of A/Virginia/ATCC3/2009 (A(H1N1)pdm09) (ATCC VR-1738; abbreviated Virg09), in a similar way as reported (74). In the pVP-NA plasmid, we replaced the entire NA-coding sequence of PR8 with that of Virg09. The H16 HA and N3 NA sequences of A/black-headed gull/Sweden/5/1999 (abbreviated Gull99) were ordered as GeneArt DNA strings from Life Technologies. To replace the sequences, high-fidelity PCR (Platinum SuperFi PCR, Invitrogen) was performed on the PR8 pVP-plasmids, and Virg09-HA and -NA plasmids prepared earlier (33), using overlapping primers. The amplified Virg09 and ordered Gull99 DNA fragments were inserted into the linearized pVP-plasmid (NEBuilder HiFi DNA Assembly Cloning Kit, New England Biolabs). To create pCAGEN expression plasmids encoding these Virg09 and Gull99 HA and NA sequences, we used high-fidelity PCR and primers extended with EcoRV and NotI sites to allow subcloning into the pCAGEN vector (provided by C. Cepko [Boston, MA] via Addgene [plasmid 11160]) (75). Specific mutations were introduced into the pVP or pCAGEN plasmids via site-directed mutagenesis with mutagenic primers and Platinum SuperFi II DNA Polymerase (Invitrogen), combined with the NEBuilder HiFi DNA Assembly Cloning Kit for pCAGEN plasmids.

### Reverse genetics

The eight-plasmid reverse genetics procedure was adapted from Martínez-Sobrido and García-Sastre (76) and described in detail before (77). To engineer the chimeric Virg09_PR8_ virus, a co-suspension of HEK293T and MDCK cells was prepared in medium with 10% FCS, transferred to a 12-well plate at 1 million cells per well of each cell line, and transfected with 4 µL Lipofectamine 2000 (Thermo Fisher Scientific) and 0.5 µg of each of the eight pVP-plasmids per well. The next day, the medium was replaced by medium with 0.3% bovine serum albumin (BSA) and 5 µg/mL tosylphenylalanylchloromethylketone (TPCK)-treated trypsin (Sigma-Aldrich). Two days later, the supernatant was collected to expand the virus in MDCK cells, using infection medium (i.e., UltraMDCK [Lonza] supplemented with 225 mg/L sodium bicarbonate, 2 mM L-glutamine, 100 U/mL penicillin-streptomycin, and 5 µg/mL TPCK-treated trypsin). The virus was collected at day 3 p.i. and stored in aliquots at −80°C. After sequencing the HA gene, virus titration was done by end-point dilution in 96-well plates containing 7,500 MDCK cells per well. After scoring the CPE at day 3 p.i., the 50% cell culture infective dose (CCID_50_) was calculated using the method of Reed and Muench.

To successfully rescue the chimeric Gull99_PR8_ virus, the above method required some modifications, i.e., transfection of a co-suspension of HEK293T and MDCK^TMPRSS2^ cells, and addition of a TMPRSS2 expression plasmid (33) to the eight pVP plasmids in the transfection mix.

### Generation of randomly mutated virus libraries and selection of acid-stable HA-mutants

To create virus libraries with random mutations in HA, we performed error-prone PCR (GeneMorph II EZClone Domain Mutagenesis Kit, Agilent) on the Virg09-HA sequence in the pVP-plasmid. Two primer sets were used (Table S4) to separately amplify two parts of the HA sequence. The PCR products served as megaprimers for the EZClone reaction. Based on a concise optimization experiment, we selected the condition of 500 ng input DNA and 30 PCR cycles to obtain a mutation frequency of approximately one mutation per sequence. In this way, six randomly mutated pVP-Virg09-HA libraries were obtained (two fragments submitted to three separate PCR reactions). These were used to conduct reverse genetics as outlined above and produce six libraries of HA-mutant viruses.

To select acid-stable mutants, the undiluted virus libraries were adjusted to pH 5.0 with citric acid, then incubated for 1 h at 37°C. After 1:4 dilution in neutral MDCK infection medium (to reduce the acidity), the viruses were added to MDCK cells seeded at 43,000 cells per well in 24-well plates, followed by medium replacement at 1 h p.i. After 3 days of incubation, the wells showing CPE were selected, and these supernatants were harvested. Following RNA extraction (QIAamp Viral RNA Mini Kit, Qiagen), the SuperScript One-Step RT-PCR System from Invitrogen was used to amplify the full HA sequence with two primer pairs: the H1R1264-H1F848 pair recommended by the WHO (https://www.who.int/teams/global-influenza-programme/laboratory-network/quality-assurance/eqa-project/information-for-molecular-diagnosis-of-influenza-virus), and another pair targeting the PR8 5′ and 3′ ends of the chimeric HA sequence. Finally, the HA fragments were submitted for Sanger sequencing by Macrogen Europe.

### Viral replication assays

To monitor viral growth kinetics, the virus was inoculated on MDCK or MDCK^TMPRSS2^ cells at a multiplicity of infection (MOI) of 200 CCID_50_ per well. The infection medium (see above) contained 5 µg/mL trypsin (for MDCK) or no trypsin (for MDCK^TMPRSS2^). The supernatants were harvested at 24, 36, 48, and 72 h p.i., then titrated in MDCK cells.

To compare replication in MDCK and Calu-3 cells, the virus was added at an MOI of 100 CCID_50_ per well, using the infection medium that contained no trypsin (Calu-3) or 5 µg/mL trypsin (MDCK). After 2 h, excess virus was removed, and the cells were further incubated at 37°C. After 3 days, immunostaining for viral nucleoprotein was conducted as described (78), followed by high-content imaging (CellInsight CX5; Thermo Scientific).

### Cell-cell fusion assays in HA-expressing cells, based on split-GFP, luminescence, or impedance

#### Split-GFP

The fluorescent assay was conducted in black-wall 96-well plates. Reverse transfection was performed at a ratio (expressed per well) of 10,000 HeLa-GFP1-10 cells, 10,000 HeLa-GFP11 cells, 50 ng HA-pCAGEN plasmid, and 0.26 µL Fugene 6. Two days later, HA was activated by 5 min incubation with 30 µg/mL TPCK-treated trypsin. Next, cell-cell fusion was induced by incubation in acidic buffer (PBS with Ca^2+^ and Mg^2+^, adjusted to pH with acetic acid) for exactly 5 min. To apply different pH conditions across wells, we used a range of buffers from pH 4.5 to 6.0, in 0.1 increments. After replacing the acidic buffer with growth medium, the plates were incubated for 24 h at 37°C. Then, fluorescent focusing beads (Bangs Laboratories) were added at 1,250 beads per well, and the plates were placed in a CellInsight CX5 high-content imaging instrument to quantify the total GFP area via the SpotDetector protocol of the imaging software. The percentage fusion was calculated by subtracting the background value at pH 7 from the GFP area at each pH tested and dividing this value by the GFP area at pH 5.0 (H1 HA) or 4.5 (H16 HA).

#### Luminescence

To obtain a quantitative readout for HA-mediated cell-cell fusion, we used the pGal5-luciferase and pGal4-VP16 plasmids (kind gift from S. Pöhlmann, Göttingen, Germany) and the transactivation setup reported by his team (79), with several modifications. On day 0, the HeLa target cells were reverse-transfected at a ratio (expressed per well) of 15,000 cells, 50 ng HA-pCAGEN plasmid, 13 ng pGal4-VP16 plasmid, and 0.26 µL Fugene 6 (Promega), then seeded in white 96-well plates. In parallel, a six-well plate was prepared with, in each well, 2 million HeLa effector cells, 500 ng pGal5-luciferase plasmid, and 2 µL Fugene 6. The next day, the effector cells were detached using a non-enzymatic cell dissociation solution (Sigma-Aldrich), resuspended, and overlaid on the target cells at 10,000 cells per well. After another 24 h, HA activation with 5 µg/mL of trypsin, followed by induction of cell-cell fusion, was conducted similarly as described above for the split-GFP assay. Finally, the cells were lysed, and luminescence was measured using the Luciferase Assay System and GloMax Navigator (both from Promega), using injector mode and 100 µL buffer per well. The percentage fusion was calculated by subtracting the background signal at pH 7 from the signal at each pH tested, then dividing this value by the one obtained at pH 5.0.

#### Impedance

The protocol was adapted from reference 39 and conducted in 16-well plates with gold microelectrodes (Agilent). On day 0, HeLa cells were reverse-transfected at a ratio of 20,000 cells, 50 ng HA-pCAGEN plasmid, and 0.26 µL Fugene 6 per well, after which the plate was placed in an xCELLigence instrument (Thermo Scientific). At 48 h post-transfection, a short baseline measurement of the CI was performed (five consecutive measurements, every 5 s). Next, the HA was exposed to 5 µg/mL trypsin, followed by 10 min incubation at pH 5.0. The formation of syncytia was followed in real-time by recording the CI value for 20 h at 2 min intervals. Data were analyzed as in reference 39. Data were analyzed as in reference 39, using the Matlab script (version R2016b, Mathworks) to normalize the raw CI values to the baseline value, followed by Graphpad Prism 10.3.1 to calculate the AUC.

### Pseudovirus entry assay

To produce murine leukemia virus (MLV)-based pseudoviruses bearing the HA and NA of interest (33), HEK293T cells were seeded in a six-well plate at 700,000 cells per well, in medium containing 0.2% FCS and 0.3% BSA. The next day, they were transfected at a ratio (expressed per well) of 6 µL Lipofectamine 2000, 750 ng MLV-packaging vector, and 1,500 ng luciferase plasmid (both kindly donated by S. Pöhlmann), combined with 1,000 ng pCAGEN-HA plasmid and 250 ng pCAGEN-NA plasmid. At day 3 post-transfection, the HA-bearing pseudovirus was activated by adding TPCK-treated trypsin at a final concentration of 80 µg/mL (unless specified otherwise). After 15 min incubation at 37°C, the same concentration of soybean trypsin inhibitor (Sigma-Aldrich) was added. In experiments assessing HA0 cleavability by TMPRSS2, −4 or −13, the pseudovirus was not activated by trypsin but, instead, 270 ng (unless specified otherwise) of a plasmid encoding TMPRSS2, −4 or −13 (33), was combined with the other four plasmids on day 0 of the production scheme. All pseudovirus stocks were harvested 3 days post-transfection and stored in aliquots at −80°C.

To determine the pseudovirus entry efficiency, 3,750 MDCK, MDCK^TMPRSS2^, or A549 cells were seeded in white half-area 96-well plates, in medium containing 0.2% FCS and 0.3% BSA. The next day, we added 10 µM zanamivir (Sigma-Aldrich), immediately followed by the pseudovirus. Entry was promoted by spinoculation for 45 min at 450 × *g* and 37°C, followed by 60 min in the incubator at 37°C. Next, the supernatant was replaced by fresh medium, and the plates were incubated for three days at 37°C. Expression of the firefly luciferase reporter was quantified using a Glomax Navigator and luciferase assay system, as described above.

### Assessment of HA0 cleavage

To assess the extent of HA0 cleavage by trypsin or TMPRSS2, a 600-µL aliquot of the pseudovirus stock was loaded on 50 µL of 20% sucrose in PBS and centrifuged for 2 h at 21,000 × *g* and 4°C. The bottom fraction was collected and lysed in RIPA buffer supplemented with protease inhibitor cocktail and EDTA (both from Thermo Fisher Scientific). The lysates were treated with PNGase F (New England Biolabs) to deglycosylate the HA0, HA1, and HA2 proteins, and optimize their separation in the Simple Western analysis. Capillary electrophoresis, antibody binding, and detection were performed according to the manufacturer’s instructions. All steps were done using default settings, except for the separation step, which was set to 35 min. Specifically, proteins were size separated on the 12–230 kDa Jess Separation Module (SMW004) and bound with primary anti-HA antibody (i.e., rabbit anti-H1 HA [SinoBiological 11055-RM05; 1/25 diluted] or goat anti-H16 HA [BEI Resources NR-34798; 1/40 diluted]), followed by secondary antibodies of detection modules DM-001 and DM-006. Next, the primary and secondary antibodies were removed using the Replex Module (RP001) to allow subsequent total protein quantification. Finally, the protein signals were visualized via Compass for Simple Western software, v.6.1.0 (ProteinSimple). To quantify the percentage cleavage, the corrected peak areas of HA1 or HA2 were divided by the sum of the corrected peak areas of HA0 plus HA1 or HA2.

### Flow cytometric analysis of HA and TMPRSS2 expression

HeLa cells were reverse transfected with HA in the same way as in the cell-cell fusion assays and seeded in a six-well plate. Two days later, the cells were washed with blank medium, then incubated with 5 µg/mL TPCK-treated trypsin to activate HA. Next, they were detached with a non-enzymatic cell dissociation solution and resuspended in medium with 10% FCS, after which they were allowed to regenerate for 1 h. Then, the cells were pelleted, resuspended in PBS, and stained with BD Horizon Fixable Viability Stain 780. A cell death control was prepared by heating at 65°C for 5 min. Subsequently, the cells were placed on ice and stained for 30 min with primary antibody (mouse anti-HA antibody C179 [Takara M145; 10 µg/mL]), followed by 30 min incubation with secondary antibody (anti-mouse IgG AF488 [Invitrogen A21131; 5 µg/mL]), with washing steps in between. All stainings were done in FACS buffer (PBS with 2% FCS). In the last step, the cells were fixed in 2% paraformaldehyde for 5 min. Data were acquired on a FACS Celesta flow cytometer (Becton Dickinson) and analyzed with FlowJo software.

To measure expression of TMPRSS2, HEK293T cells were transfected with the TMPRSS2 plasmid in the same way as done for the pseudovirus production. At 72 h post-transfection, the cells were detached with non-enzymatic cell dissociation solution and resuspended in medium with 10% FCS, in which they were allowed to regenerate for 1 h. Afterward, the staining procedure and flow cytometric analysis were conducted as described above. The primary antibody was rabbit anti-TMPRSS2 antibody from Abcam (ab280567; 0.5 µg/mL), and the secondary antibody was anti-rabbit IgG from Cell Signaling Technology (4414S; 1 µg/mL).

### Generation and alignment of consensus cleavage loop sequences

For each HA subtype, 50–1,000 sequences per clade (or per genotype in case of H5 clade 2.3.4.4.b) were aligned to generate clade consensus sequences and derive subtype consensus sequences. For subtypes with fewer than 50 sequences in the GISAID database (80), we included all available sequences, and for H19 HA, we aligned the three published sequences (81, 82). In the final step, we aligned the consensus cleavage loop sequences across all 19 subtypes.

### HA models and graphics

To study the stabilizing mutations and cleavage loop structures on a molecular level, SWISS-MODEL (83) was used to model the HA sequence of our Virg09 strain on several cryo-EM and crystal structures, i.e., PDB 3M6S (46), PDB 6Y5K (5), and PDB 1HTM (47). For the structure of H16 HA0, we used PDB 4F23 (15). The cleavage loops were modeled with AlphaFold (45), and the graphics were made with ChimeraX (84).

### Schematics, data analysis, and statistics

Graphical schemes were created using BioRender (Naesens, L. [2025] https://BioRender.com/z9ws7tt) and edited in PowerPoint and Photoshop. All graphs were created with GraphPad Prism 10.3.1 software, which was also used for statistical analysis. Statistical significance is shown as: ****P ≤ 0.0001, ***P ≤ 0.001, **P ≤ 0.01, and *P ≤ 0.05; ns: not significant.

## SUPPLEMENTAL MATERIAL

Supplemental Material spectrum.04123-25-s0001.pdf Tables S1–S4 and Figures S1–S6.

ASM does not own the copyrights to Supplemental Material that may be linked to, or accessed through, an article. The authors have granted ASM a non-exclusive, world-wide license to publish the Supplemental Material files. Please contact the corresponding author directly for reuse.
