# Supplementary material for: New determinant of influenza hemagglutinin cleavability identified through random mutagenesis for acid-stabilizing mutations
Source: Microbiol Spectr. 2026 Jun 15;14(7):e04123-25. doi: 10.1128/spectrum.04123-25 (PMC13340207; doi:10.1128/spectrum.04123-25)
Supplement: Supplemental Material — Tables S1–S4 and Figures S1–S6. [file spectrum.04123-25-s0001.pdf]

New determinant of influenza hemagglutinin cleavability identified through random mutagenesis for acid-stabilizing mutations

S. Rimaux, M. F. Oliva, C. Mestdagh, R. Van Berwaer, J. Chen, L. Schurmans, G. Schoofs, B. Vanmechelen, J. Stroobants, M. Jacquemyn, M. Laporte, P. Maes, K. Vermeire, K. Das, A. Stevaert, L. Naesens

Supplemental material

Table S1. H3-numbering for the investigated residues.

HA0 numbering from the mature protein	H3-numbering[a]
I79	$I87_1$
Q104	$Q109_1$
G202	$G205_1$
K209	$K212_1$
K211	$K214_1$
T241	$T244_1$
D346	$D19_2$
E374	$E47_2$
I383	$I56_2$
K454	$K127_2$

[a]The suffix denotes whether the residue is located in the HA1 or HA2 subunit.

Table S2. Rescue of Virg09$_{PR8}$ viruses containing stabilizing HA mutations.

Mutant	Log_{10} $CCID_{50}$/mL[a]
WT	5.60 ± 0.16
I79V	5.17 ± 0.39
K211T	5.68 ± 0.26
T241S	5.51 ± 0.36
E374K	5.59 ± 0.36
K454N	5.70 ± 0.25
Triple	5.94 ± 0.24
Quadruple	5.52 ± 0.17
Quintuple	5.42 ± 0.34

[a]Virus titers of the supernatants, determined in MDCK cells.
Data are the mean ± SEM (N=4).

Table S3. Prevalence[a] of extra mutations acquired after four passages of the HA-mutant viruses in MDCK cells.

HA-mutant	Extra HA mutation upon passaging	Neutral pH	Low pH
I79V	K119N	78%	73%
T241S	K209E	31%	0.16%
E374K	A9T	14%	0.05%
	N398H	57%	15%
Quadruple	N294S	17%	13%
	L400P	26%	18%
	D412N	41%	37%
Quintuple	R403G	32%	10%

[a]The data show the prevalence (in %) of the extra mutations after the viruses were passaged three times, then incubated for 1 h in medium at either neutral pH or pH ~5, and finally grown for a fourth passage. The virus populations were then analysed by Nanopore sequencing.

The result is from two experiments, conducted in quadruplicate.

Table S4. Primer sets used for random mutagenesis on the coding sequence of Virg09-HA.

Target	Forward primer	Reverse primer
$Virg09_{ecto}$-HA-Fragment-1	5'-GCAAACCTACTGGTCCTGTTAT-3'	5'-TGGCCCCAAATAGGCCT-3'
$Virg09_{ecto}$-HA-Fragment-2	5'-ATGTCCCGTCTATTCAATCTAGA-3'	5'-AGTTGAGTAGATCGCCAGAATC-3'

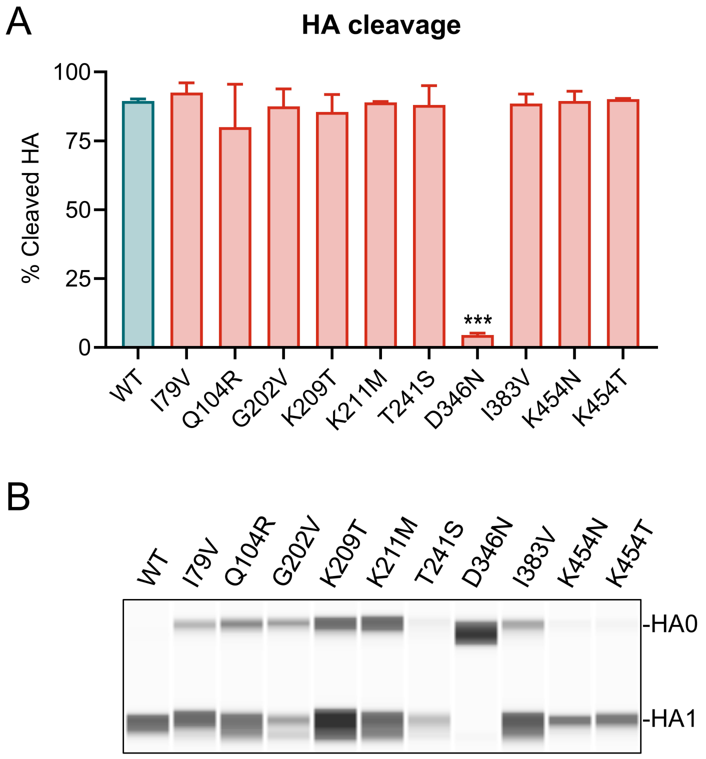

Figure S1. HA cleavage profile of WT and selected HA mutants. (A) The ten mutations, selected by random mutagenesis and low pH treatment, were engineered in H1N1 pseudoviruses which were treated with trypsin, then submitted to Simple Western analysis to quantify the % cleaved HA. Only the D346N mutant showed a significant difference versus WT (Brown-Forsythe and Welch ANOVA followed by post-hoc comparisons using Dunnett's T3 test). (B) Representative Wes image showing the HA cleavage pattern of WT and mutant pseudoviruses after trypsin treatment.

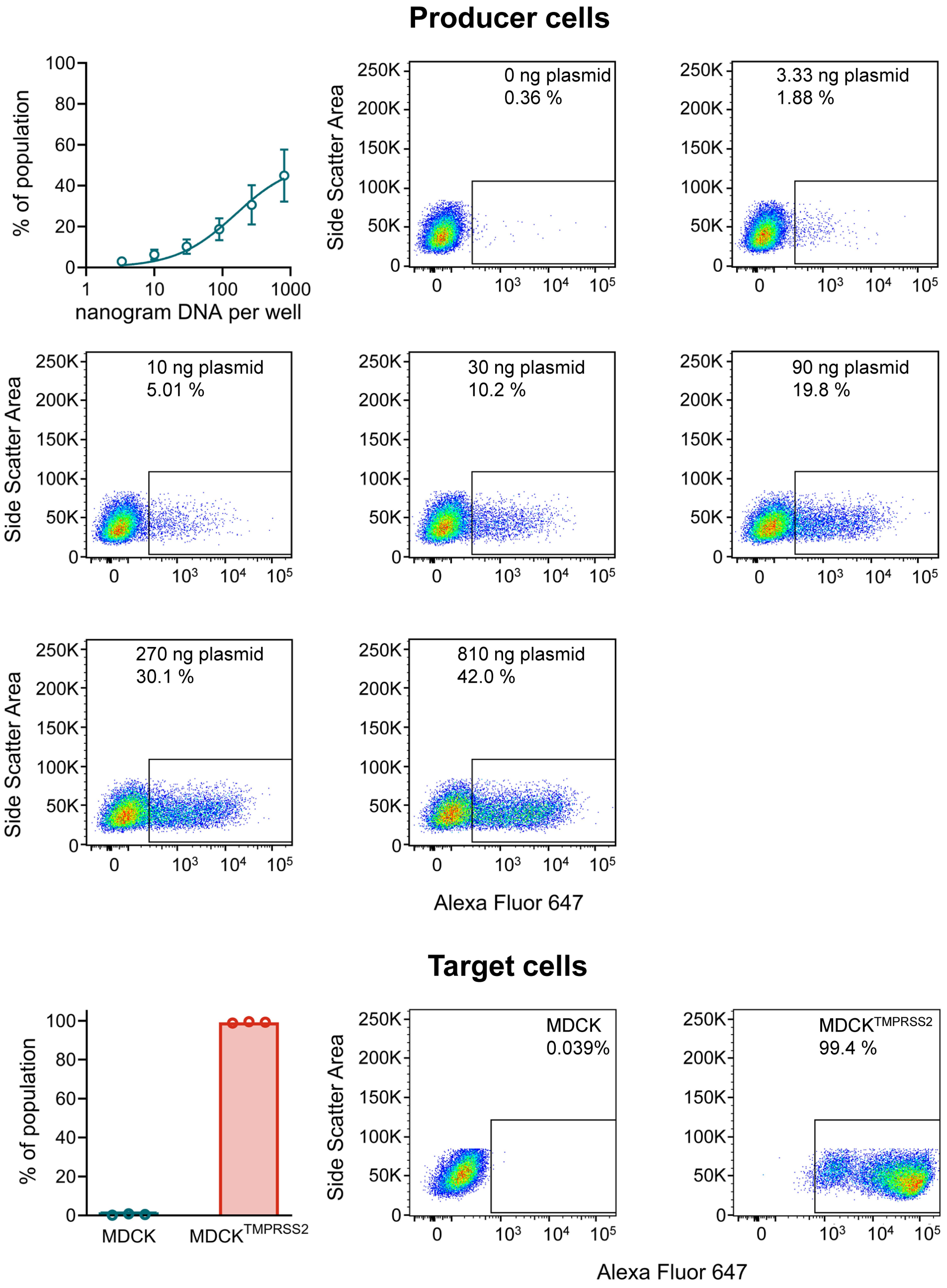

Figure S2. Flow cytometry to verify robust and plasmid concentration-dependent TMPRSS2 expression. The data show the TMPRSS2-positivity of the cell population, assessed in either HEK293T producer cells transfected with different amounts of TMPRSS2 plasmid (xy graph and scatterplot on top) or the stably transduced MDCK^{TMPRSS2} target cells (bar graph and scatterplots on the bottom). The xy graphs show the mean ± SEM (N=3). In the bar graphs, each data point is shown and the bar represents the mean. The scatterplots are taken from one representative experiment.

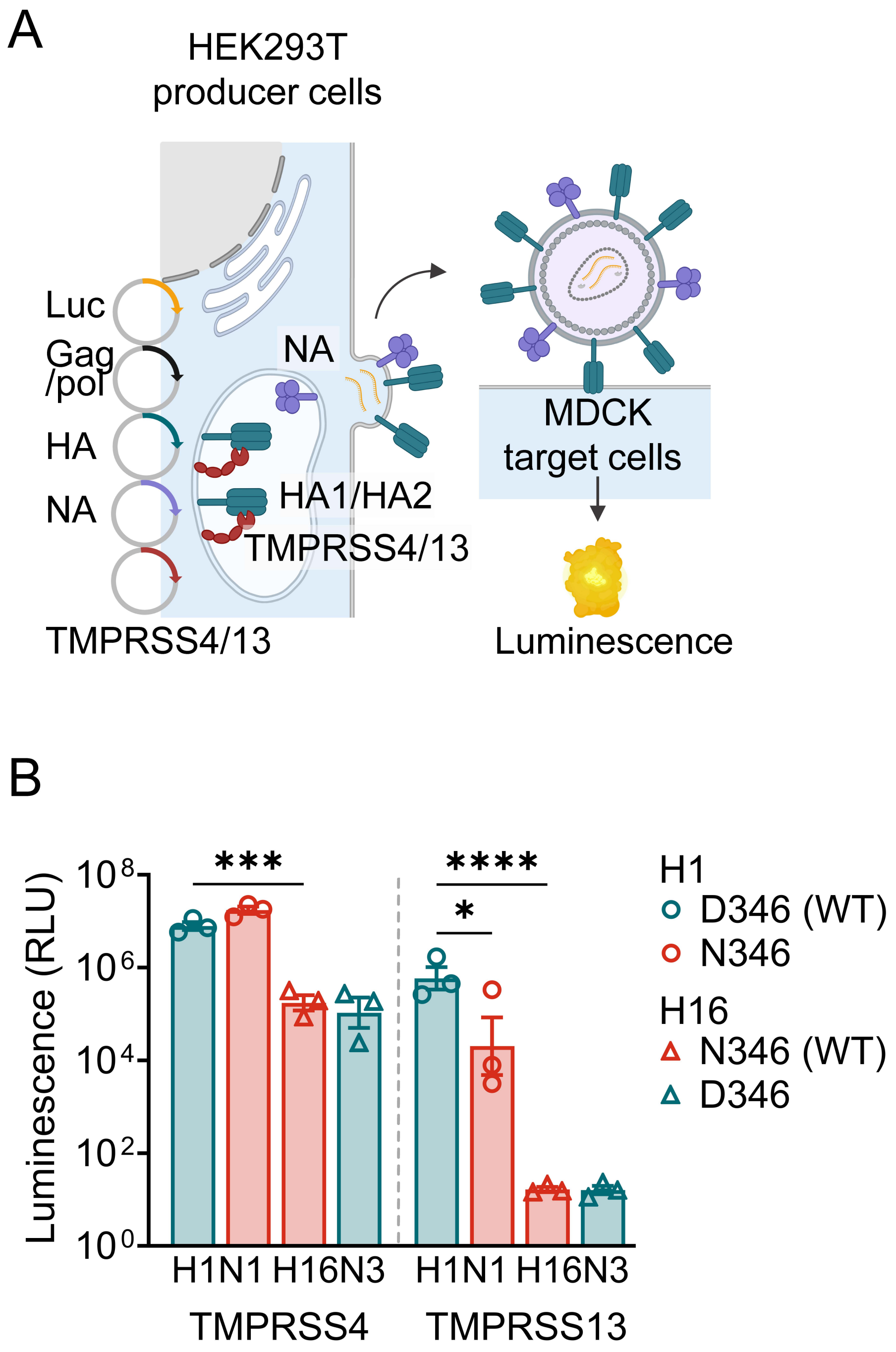

Figure S3. Cell entry capacity of pseudovirions released from HA- and TMPRSS4- or TMPRSS13- co-transfected cells. (A) Setup of the luciferase-based pseudovirus entry assay. TMPRSS4 or TMPRSS13 plasmid was added to the transfection mix to activate the H1N1 or H16N3 pseudovirus during production. The viruses were transduced in MDCK cells, followed by luciferase readout after 72 h. (B) Individual and mean RLU values ± SEM (N=3). Statistical significance was analyzed by ordinary one-way ANOVA with Šídák's multiple comparisons test.

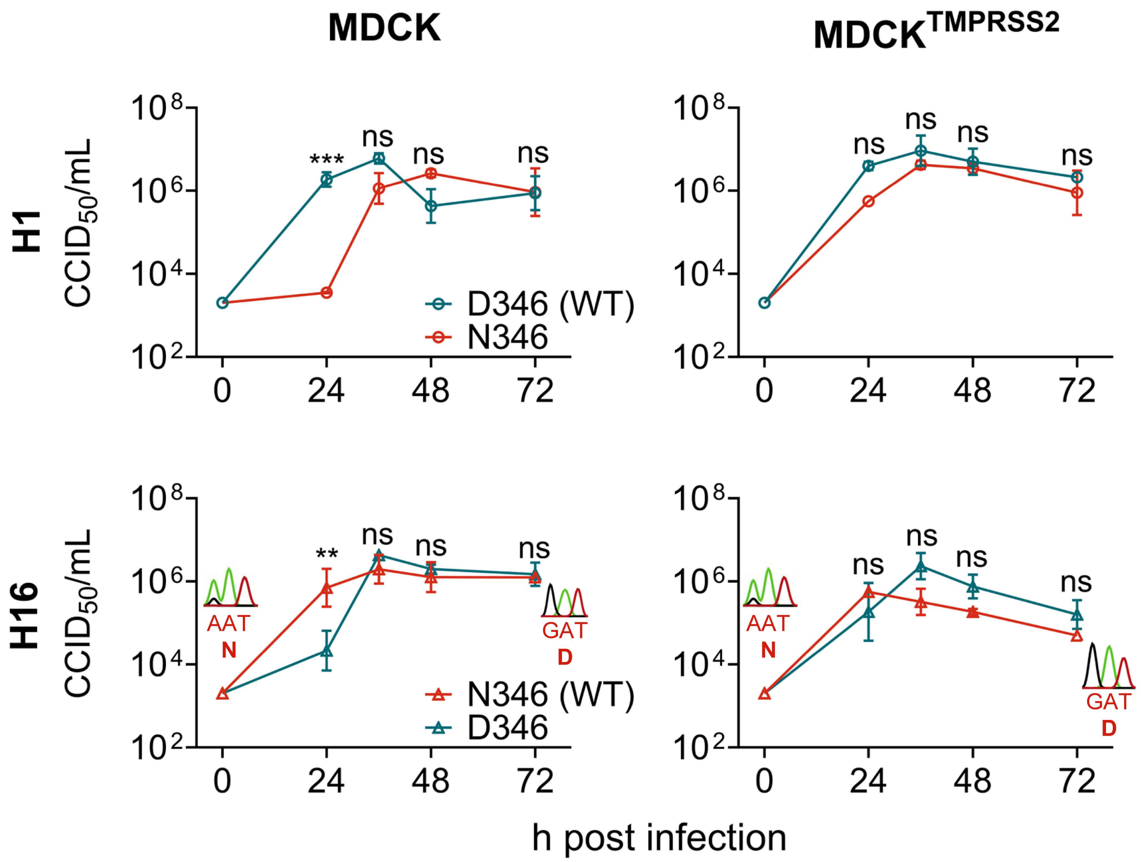

Figure S4. Growth kinetics of WT and mutant Virg09$_{PR8}$ (H1N1) and Gull99$_{PR8}$ (H16N3) viruses in MDCK and MDCK^{TMPRSS2} cells. The cells were infected (MOI: 200 $CCID_{50}$) and supernatants were collected at 24, 36, 48 and 72 h p.i., followed by virus titration in MDCK cells. Data points are the mean ± SEM (N=3). For each time point, significance is shown for the difference between mutant and WT (multiple unpaired t-tests, with Holm-Šídák's correction for multiple comparisons). Next to the H16N3 graphs, a zoom is shown of the HA sequence. Whereas the virus stock carried a mixed population of N346/D346, the D346 form dominated at 72 h p.i. in MDCK and MDCK^{TMPRSS2} cells.

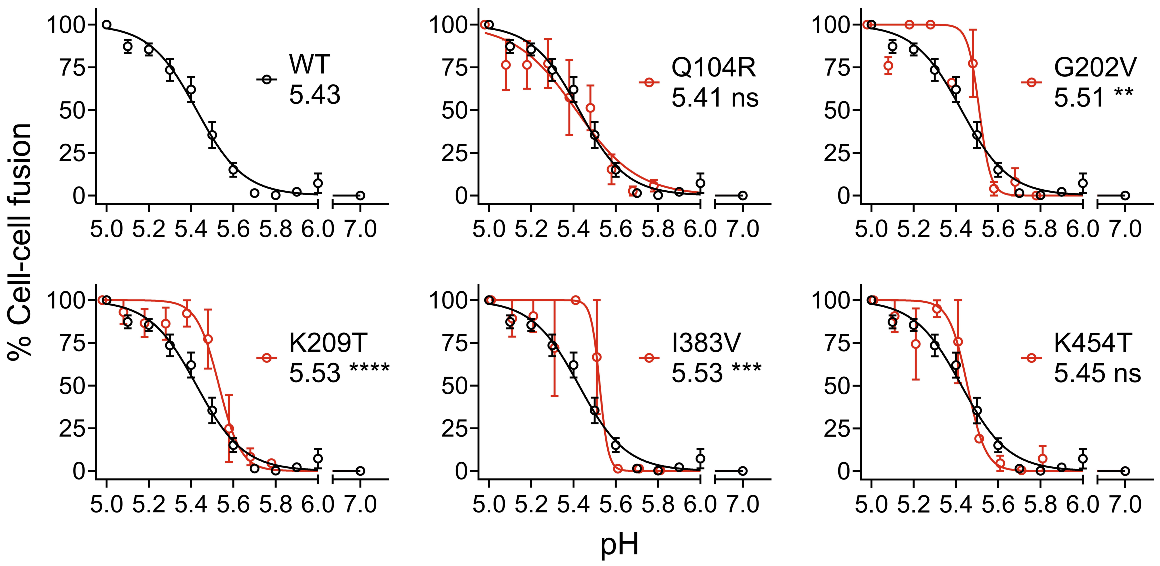

Figure S5. pH profiles of other mutations obtained after random mutagenesis of H1 HA. pH profiles obtained from cell-cell fusion assays with WT HA (Virg09; black curve that is shown in each panel) and five mutants (curves shown in red). The Y-axis shows the % cell-cell fusion relative to the pH 5.0 condition, calculated by dividing the luminescence signal at each pH by the one at pH 5.0 after background subtraction. The legend shows the fusion pH, defined as the pH where fusion was 50% relative to pH 5.0. Statistical significance is shown for the difference in fusion pH between mutant and WT (extra sum of squares F test of best-fit value). Data are the mean ± SEM (N=3-4).

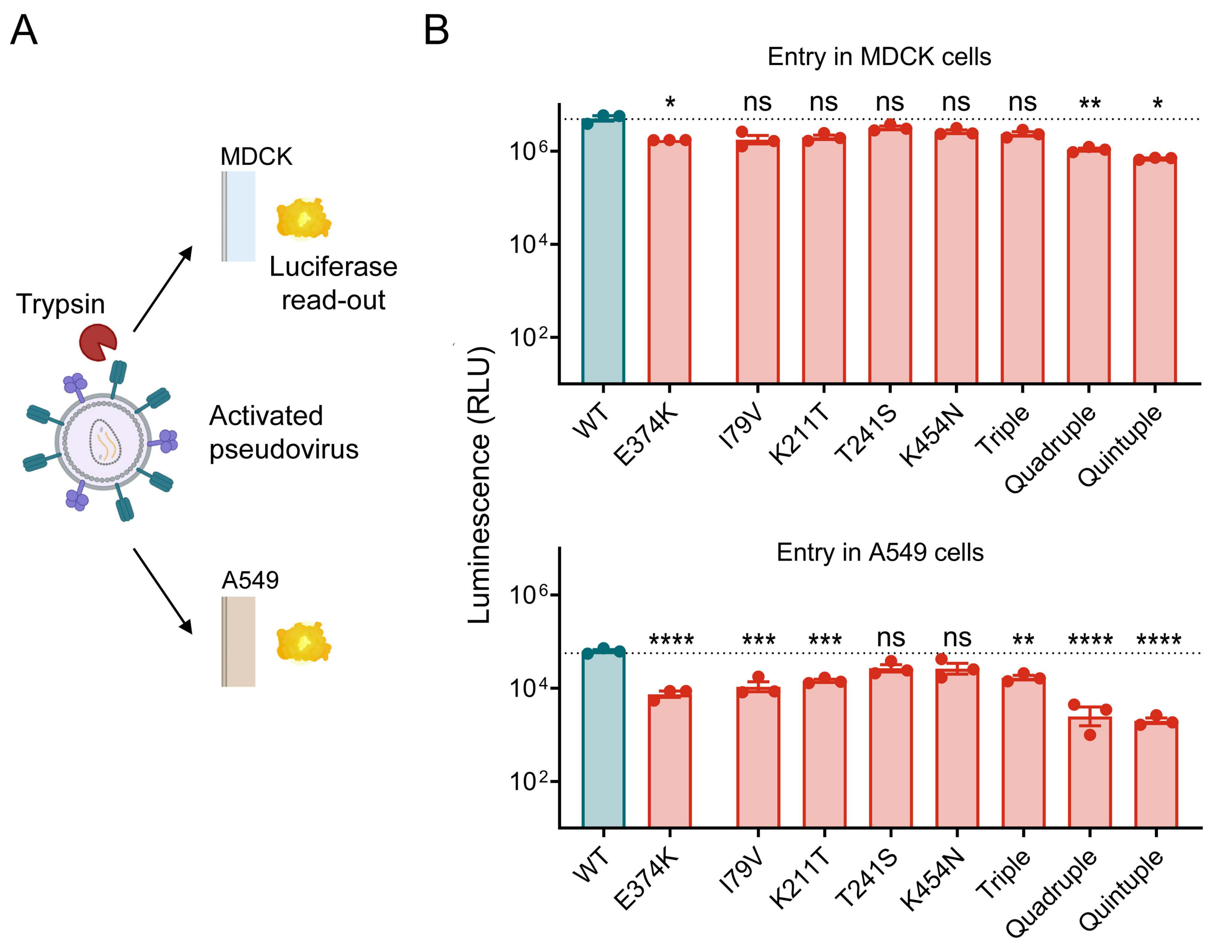

Figure S6. Entry of HA-mutant H1N1 pseudoviruses into MDCK and A549 cells. (A) HA0-bearing pseudoviruses were activated with trypsin and transduced into the cells, followed by luciferase readout at 72 h. (B) Entry in MDCK cells (top) and A549 cells (bottom) for WT and mutants [triple = I79V + K211T + K454N; quadruple = triple + E374K; quintuple = quadruple + T241S]. Data show individual and mean RLU values ± SEM (N=3). Statistical analysis for mutants versus WT, based on RM one-way ANOVA with Dunnett's corrections for multiple comparisons.
